# *Eucalyptus globulus* Bark Extract and Hinokitiol in Hybrid Anti-Sapstain Formulations with Reduced Chlorothalonil, Carbendazim and Copper-8-quinolinolate Loads

**DOI:** 10.3390/molecules31193503

**Published:** 2026-10-01

**Authors:** Gastón Bravo-Arrepol, Solange Torres, Victor Ferrer, Danilo Escobar-Avello, Moisés Vásquez, Vicente Hernández, Gustavo Cabrera-Barjas, Claudia Pérez, Cecilia Fuentealba

**Affiliations:** 1 Facultad de Ciencias, Universidad San Sebastián, Campus Las Tres Pascualas, Lientur 1457, Concepción 4080871, Chile; 2 Laboratorio de Química de Productos Naturales, Departamento de Botánica, Facultad de Ciencias Naturales y Oceanográficas, Universidad de Concepción, Barrio Universitario S/N, Concepción 4070386, Chile; 3 Facultad de Medicina Veterinaria y Agronomía, Instituto de Ciencias Naturales, Universidad de Las Américas, Campus El Boldal, Av. Jorge Alessandri 1160, Concepción 4090940, Chile; 4 Unidad de Desarrollo Tecnológico, Universidad de Concepción, Av. Cordillera 3624, Parque Industrial Coronel, Coronel 4191996, Chile; 5 Centro Nacional de Excelencia para la Industria de la Madera (CENAMAD)—ANID BASAL FB210015, Pontificia Universidad Católica de Chile, Av. Vicuña Mackenna 4860, Santiago 7820436, Chile; 6 Departamento de Manejo de Bosques y Medio Ambiente, Facultad de Ciencias Forestales, Universidad de Concepción, Victoria 631, Concepción 4070386, Chile; 7 Centro de Biotecnología, Universidad de Concepción, Barrio Universitario S/N, Concepción 4070386, Chile; 8 Escuela de Nutrición y Dietética, Facultad de Ciencias de la Rehabilitación y Calidad de Vida, Universidad San Sebastián, Campus Las Tres Pascualas, Lientur 1457, Concepción 4080871, Chile

**Keywords:** sapstain fungi, copper-8-quinolinolate, acute aquatic toxicity, tropolone, ellagic acid derivatives, factorial design, forestry residues

## Abstract

The general objective of this study was to evaluate *Eucalyptus globulus* bark extract as a candidate component of wood preservatives with a reduced load of synthetic biocides. Extraction conditions were screened using a 2^3^ full factorial design with a center point, identifying 75 °C, 60% ethanol and 1 h as the highest-yielding conditions (5.94%) within the tested ranges. In four-week laboratory wood-block assays, the extract alone did not exceed 40% inhibition of wood-staining fungi, whereas hinokitiol reached complete inhibition at 0.4%. Combining the extract (5%) with hinokitiol (0.4%) achieved 95–100% inhibition, similar to the synthetic reference; because hinokitiol alone reached complete inhibition at this concentration, the extract’s contribution could not be isolated, and formulations in which the extract or hinokitiol was used instead of chlorothalonil and carbendazim alongside a copper-8-quinolinolate product retained 97–100% inhibition at half, and 90–93% at a quarter, of the copper-product dose (87% and 93% reductions in synthetic active ingredients), although the copper product alone was also fully effective at 2% and the contribution of the natural components could not be isolated at that dose. On a per-mass basis, the extract (48 h LC_50_ 27,500 ppm) was approximately 710-fold less acutely toxic than the copper-8-quinolinolate product used as synthetic reference (38.71 ppm) in *Daphnia magna* bioassays. On the basis of its composition and the literature, the extract may contribute multi-target antifungal mechanisms, including metal chelation, enzyme inhibition and membrane disruption, that could complement the more stable action of hinokitiol; these mechanisms were not measured here. This work supports eucalyptus bark extract, a residue that is abundant in Chile and in other eucalypt-pulp-producing countries, as a candidate component of reduced-synthetic (hybrid) wood-protection formulations; the aquatic toxicity of the final formulations was not tested, and leaching, penetration and field-durability testing are required before practical or environmental benefits can be claimed.

## 1. Introduction

Wood and products made from wood are indispensable in the construction and manufacturing industries around the world and are highly regarded for their renewability and structural properties. However, colonization of wood by wood-staining and surface-mold fungi such as *Ophiostoma* spp., *Diplodia* spp., and *Trichoderma* spp. leads to substantial economic losses because of discoloration of the sapwood, which compromises the aesthetic quality and market value of the wood even though these fungi do not substantially degrade its structural cell-wall polymers [1,2,3]. The prevention of fungal staining is a crucial problem in wood preservation.

Traditional wood preservatives, especially chromated copper arsenate (CCA), have afforded good levels of protection against wood destroying organisms at serious environmental and public health cost. The arsenic component of CCA is a known human carcinogen and the leaching of toxic metals from treated wood into soil and water has caused significant concern about contamination of the ecosystem as well as humans [4,5]. As a result, regulatory frameworks have become increasingly strict. The European Union’s Biocidal Products Regulation (BPR) No 528/2012 has set stringent requirements on biocidal active substances used for wood preservation, and several fungicides containing azoles have been limited or prohibited because of their high toxicity to aquatic organisms [6,7,8]. This changing regulatory situation has led to a pressing demand for alternative preservatives for wood that are both effective and environmentally benign.

Plant extracts and other naturally derived compounds have therefore attracted growing attention as candidate replacements for conventional biocides. However, a fundamental challenge restricts their use as stand-alone preservatives: moderate efficacy, attributable in part to the capacity of many wood-inhabiting fungi to oxidise, conjugate or otherwise detoxify phenolic compounds. White-rot fungi possess arsenals of oxidative and conjugating enzymes, including laccases, cytochrome P450 monooxygenases and glutathione S-transferases, that degrade or conjugate bioactive compounds in natural extracts, thereby neutralizing their antifungal effects. Although these enzyme systems are most elaborate in lignolytic decay fungi, sapstain fungi such as *Ophiostoma* also express phenol-oxidising and detoxifying activities that can limit the efficacy of single natural compounds.

*Eucalyptus globulus* bark, a Forestry and Pulp industry byproduct, is an abundant and underestimated source of bioactive compounds with phenolic acid (gallic acid, ellagic acid), flavonoid glycoside, condensed tannins, and lipophilic terpenoids (β-sitosterol, α-amyrin) with known antioxidant and antimicrobial properties [9]. To overcome the limitations of the use of natural extracts exclusively, this research examines the strategic use of hinokitiol (β-thujaplicin), a naturally occurring tropolone that underlies the exceptional decay resistance of western redcedar (*Thuja plicata*) and hinoki cypress (*Chamaecyparis obtusa*) [10,11,12]. Hinokitiol has significant antifungal activity due to metal ions (Fe^2+^, Cu^2+^) chelation and has a higher stability against enzymatic degradation than many phenolic compounds making it a more persistent antifungal agent [13,14,15].

The rationale for development of hybrid formulations of a combination of eucalyptus bark extract and hinokitiol is based on multi-target antifungal action. The complex mixture of phenolics and terpenoids found in the extract of the bark of this plant exert multiple modes of action (metal chelation, direct enzyme inhibition, membrane disruption, free radical scavenging) that work collectively to put pressure on fungal metabolism in several places [15]. When combined with hinokitiol, with its strong metal-chelating ability and enzymatic stability, the resulting formulation may exert additive or synergistic effects that overwhelm the fungal detoxification mechanisms to enhance overall efficacy and minimize the need for highly toxic synthetic biocides to a great extent.

The general objective of this study was to evaluate *E. globulus* bark extract as a candidate component of anti-sapstain wood-preservative formulations with a reduced load of synthetic biocides. The specific objectives were: (1) to screen the extraction conditions of *Eucalyptus globulus* bark with a 2^3^ factorial design in order to identify the most favorable condition within the tested ranges, (2) to characterize the lipophilic and phenolic profile of the selected extract by gas chromatography–mass spectrometry (GC-MS) and ultra-high-performance liquid chromatography–mass spectrometry (UHPLC-MS), (3) to determine the in vitro and in wood antifungal efficacy against wood-staining fungi, (4) to develop the hybrid formulations of eucalyptus bark extract with hinokitiol and with commercial biocides, and evaluate their antifungal efficacy, (5) to assess the acute aquatic toxicity of the extract by *Daphnia magna* bioassays. We hypothesized that hybrid formulations combining *Eucalyptus globulus* bark extract with hinokitiol, or with a reduced dose of a copper-8-quinolinolate product, can provide anti-sapstain efficacy similar to that of the conventional industrial mixture (copper-8-quinolinolate, chlorothalonil and carbendazim; the latter two are no longer approved or are restricted in several jurisdictions, including the European Union, because of human-health and environmental hazards [6,7]) in laboratory wood-block assays while substantially reducing the load of synthetic active ingredients. The acute aquatic toxicity of the extract and of the copper-8-quinolinolate product was measured; that of the final formulations was not.

## 2. Materials and Methods

### 2.1. Plant Material and Reagents

*Eucalyptus globulus* bark was obtained as an industrial by-product of managed commercial plantations operated by Forestal Collicura (Santa Juana, Biobío Region, Chile), the same source used in a previous study on *E. globulus* bark valorisation by our group [16]. Because the material was sourced from commercial plantation stock of a cultivated species rather than from wild or protected populations, no collection permits or access-and-benefit-sharing procedures under the Nagoya Protocol were required, and no herbarium voucher was deposited; the species identity is that of the certified plantation stock. The material was air-dried at room temperature until constant weight, ground into a fine powder (particle size < 1 mm), and stored in sealed bags at 4 °C until use. The moisture content of the air-dried powder was not determined; extraction yields are therefore expressed on an air-dry bark basis. All solvents used for extraction and analysis were of analytical grade. Hinokitiol (β-thujaplicin, >98% purity) was purchased from a commercial supplier. Three commercial anti-sapstain products used by the collaborating sawmill, designated here commercial products 1–3 (CP1, copper-8-quinolinolate, 5.2% *w*/*w*; CP2, chlorothalonil, 72% *w*/*v*; CP3, carbendazim, 50% *w*/*v*), and a fourth commercial product used as positive control in the diffusion assay (CP4, copper-8-quinolinolate, 5.91% *w*/*w*) were used as supplied (Section 2.5 and Section 2.6); trade names are not given, and the products are identified throughout by their declared active ingredients and contents. DPPH (2,2-diphenyl-1-picrylhydrazyl), ABTS (2,2′-azino-bis(3-ethylbenzothiazoline-6-sulfonic acid)), Folin–Ciocalteu reagent, and gallic acid were obtained from Sigma-Aldrich (St. Louis, MO, USA).

### 2.2. Experimental Design and Screening of the Extraction Conditions

Extraction conditions were screened using a 2^3^ full factorial design with a center point [17]. The two-level, three-factor design, with a center point, was implemented using Design-Expert^®^ software (Version 11, Stat-Ease, Inc., Minneapolis, MN, USA). The independent variables were temperature (X1: 75 and 95 °C), ethanol concentration (X2: 40 and 60% *v*/*v*), and extraction time (X3: 1 and 3 h), with the center point at 85 °C, 50% *v*/*v* and 2 h. This design resulted in nine experimental runs (STD1–STD9; Table S2), each conducted in duplicate. A full 2^3^ factorial resolves the three main effects and all of their interactions, while the center point provides a check for curvature within the experimental domain [18].

For each run, 62.5 g of powdered eucalyptus bark was mixed with 500 mL of the ethanol–water solvent at the composition prescribed by the design (40, 50 or 60% *v*/*v* ethanol in distilled water) in a 1 L glass reactor equipped with a condenser and magnetic stirrer. The mixture was heated to the target temperature and maintained for the specified time. After extraction, the mixture was filtered under vacuum through Whatman No. 1 filter paper, and the solvent was removed from the filtrate using a rotary evaporator at 45 °C. The resulting crude extract was lyophilized to obtain a dry powder. The crude extraction yield was calculated as the mass of lyophilized crude extract relative to the mass of air-dried bark. The polynomial model fitted to the responses, in coded variables, was (Equation (1)):

Y = β_0_ + Σ β_i_X_i_ + Σ β_ij_X_i_X_j_ + β_123_X_1_X_2_X_3_ (1)

where Y is the predicted response, β_0_ is the intercept, and β_i_, β_ij_ and β_123_ are the main-effect and interaction coefficients of the coded variables; the center point was used to test for curvature.

### 2.3. Extract Fractionation and Carbohydrate Removal

To enhance the concentration of bioactive compounds and remove interfering carbohydrates, the crude extracts underwent a methanol precipitation fractionation process [19]. The lyophilized crude extract was redissolved in distilled water and the solution was treated with cold methanol (4:1 *v*/*v* methanol:extract solution) at 4 °C for 24 h to precipitate polysaccharides and other high molecular weight carbohydrates. The mixture was centrifuged at 4000 rpm for 20 min, and the supernatant containing phenolic and terpenoid compounds was collected. The methanol-soluble fraction was concentrated under vacuum at 40 °C and lyophilized to obtain a dry powder, which was stored at −20 °C until analysis [20,21].

#### 2.3.1. Quantification of Total Carbohydrates (Phenol-Sulfuric Acid Method)

The total carbohydrate content in both crude and fractionated extracts was determined using the phenol-sulfuric acid method [22,23]. The assay was carried out in 96-well microplate format as described by Masuko et al. [23]: 50 μL of extract solution (1 mg mL^−1^ in distilled water) or of glucose standard was dispensed into each well, 150 μL of concentrated sulfuric acid (95–97%) was added rapidly and 30 μL of 5% (*w*/*v*) phenol solution was then added; the plate was incubated for 5 min at 90 °C in a water bath and cooled to room temperature (22–25 °C) for 5 min before reading. The absorbance was measured at 490 nm using a SPECTROstar Nano microplate reader (BMG LABTECH, Ortenberg, Germany; spectral range 220–1000 nm). A calibration curve was prepared using D-glucose dissolved in distilled water as the standard (0–100 μg mL^−1^), and results were expressed as glucose equivalents per gram of extract (% *w*/*w*). The carbohydrate removal efficiency was calculated as: [(Carbohydrate content in crude extract − Carbohydrate content in fractionated extract)/Carbohydrate content in crude extract] × 100.

#### 2.3.2. Quantification of Total Phenolic Content (Folin–Ciocalteu Method)

The total phenolic content was determined using the Folin–Ciocalteu colorimetric method [24,25]. The assay was carried out in 96-well microplate format: 20 μL of extract solution (1 mg mL^−1^) was mixed with 100 μL of Folin–Ciocalteu reagent (diluted 1:10 with distilled water) and allowed to react for 5 min at room temperature (22–25 °C). Then, 80 μL of sodium carbonate solution (7.5% *w*/*v*) was added to neutralize the mixture and initiate color development (final volume 200 μL). The plate was incubated in darkness at room temperature for 30 min. The absorbance was measured at 765 nm using the same microplate reader against a blank prepared with the same solvents. A calibration curve was constructed using gallic acid as the standard (0–500 μg mL^−1^), and results were expressed as milligrams of gallic acid equivalents per gram of extract (mg GAE g^−1^). All measurements were performed in triplicate.

### 2.4. Antioxidant Activity Assessment

The antioxidant capacity of the fractionated extracts was evaluated using three complementary spectrophotometric assays performed in 96-well microplates on the SPECTROstar Nano reader described above. Each of the two independent extractions of every run was assayed in technical triplicate; the values reported are the means of the two extractions, which were the experimental units for statistical comparison (Section 2.9).

#### 2.4.1. DPPH Radical Scavenging Activity

The DPPH assay was performed according to the method described by Brand-Williams et al. [26], with slight modifications. In each well, 25 μL of extract solution (1 mg mL^−1^) or of gallic acid standard (1–40 mg L^−1^) was mixed with 250 μL of a methanolic solution of DPPH (120 μM; final volume 275 μL). The absorbance was measured at 517 nm after 30 min of incubation in the dark at room temperature (22–25 °C). The scavenging activity was expressed as mg of Gallic Acid Equivalents per gram of dry extract (mg GAE g^−1^).

#### 2.4.2. ABTS Radical Scavenging Activity

The ABTS assay was conducted following the procedure of Re et al. [27]. The ABTS radical cation (ABTS^•+^) was generated by mixing equal volumes (5 mL + 5 mL; 10 mL in total) of 7 mM ABTS stock solution and 2.45 mM potassium persulfate solution and storing the mixture in darkness for 12–16 h. The working solution was diluted to an absorbance of 0.7 ± 0.02 at 734 nm. Extract solution (1 mg mL^−1^) or gallic acid standard (1–20 mg L^−1^; 25 μL) was then added to 225 μL of the ABTS^•+^ working solution (final volume 250 μL), and the absorbance was recorded at 734 nm after 30 min. Results were expressed as mg GAE g^−1^.

#### 2.4.3. Ferric Reducing Antioxidant Power (FRAP)

The FRAP assay was carried out as described by Benzie and Strain [28]. The FRAP reagent was prepared by mixing 300 mM acetate buffer (pH 3.6), 10 mM 2,4,6-tripyridyl-s-triazine (TPTZ) in 40 mM HCl, and 20 mM FeCl_3_·6H_2_O in a 10:1:1 (*v*/*v*/*v*) ratio (8.3, 0.83 and 0.83 mL, respectively, for 10 mL of reagent). The reagent was warmed to 37 °C in a thermostatic water bath before use. Extract solution (1 mg mL^−1^) or gallic acid standard (1–200 mg L^−1^; 25 μL) was added to 250 μL of FRAP reagent (final volume 275 μL), and the absorbance of the resulting blue-colored complex was measured at 593 nm after 30 min of incubation at 37 °C. The antioxidant power was expressed as mg GAE g^−1^.

### 2.5. In Vitro Antifungal Activity

The antifungal activity of the extracts was tested against wood-staining fungi (*Trichoderma* sp., *Diplodia seriata* and *Ophiostoma* sp.) by disc- and agar-diffusion methods. The test fungi were sapstain isolates from the culture collection of the Centro de Biotecnología, Universidad de Concepción (*Trichoderma* sp. strain TRCCP_01, *Ophiostoma* sp. strain OPCCP_01 and *Diplodia seriata* strain DSCCP_01, all isolated from Pinus radiata wood or wood products), maintained on malt-extract agar; the isolates were identified by the culture collection by morphological and molecular methods at the time of isolation (records held by the Centro de Biotecnología, UdeC). The sequences obtained at isolation were not deposited in a public repository, so no accession numbers are available, which is acknowledged as a limitation. Sterile filter-paper discs (Whatman No. 3; 6 mm diameter; Whatman International Ltd., Maidstone, UK) were impregnated with 20 μL of extract solution prepared in 70% (*v*/*v*) ethanol at various concentrations (50–1000 mg mL^−1^, corresponding to 1–20 mg of extract per disc) and placed on 2% malt-extract agar plates, which were then inoculated with a mycelium disc of each fungus; three plates were used per condition (*n* = 3). The solvent used to prepare the extracts served as negative control, and two commercial anti-sapstain preparations were used as positive controls: CP4 (copper-8-quinolinolate, 5.91% *w*/*w*; Section 2.1) and the CP1–CP3 industrial mixture described in Section 2.6. The plates were incubated at 20 °C and 60% relative humidity for 6–7 days. Inhibition of mycelial growth was calculated as % inhibition = [(dc − dt)/dc] × 100, where dc and dt are the mean colony diameters (mm) in the control and treated plates, respectively. At 500–1000 mg mL^−1^ the preparations were viscous suspensions rather than true solutions, so their diffusion into the agar was limited. Because standardized antifungal-susceptibility guidelines such as those of the CLSI were developed for clinical yeasts and molds rather than for wood-inhabiting fungi, these diffusion assays were used only as a preliminary qualitative screen; quantitative efficacy was subsequently determined on wood specimens (Section 2.6).

### 2.6. Wood Specimen Testing and Formulation Development

The preservative efficacy of the extract, hinokitiol and their formulations with commercial biocides was evaluated on unseasoned *Pinus radiata* D. Don sapwood specimens (7 mm × 20 mm × 70 mm, thickness × width × length; moisture content above 40%) cut from knot-free, stain-free sapwood battens of a single tree and of the same growth-ring region, and conditioned and tested according to the ASTM D4445-10 standard laboratory method [29]. Each treatment was applied to ten replicate specimens, which were dipped for 10 s in the treatment solution and aired for 24 h under sterile conditions before inoculation. Treatment solutions contained the lyophilized fractionated extract at the stated concentration (% *m*/*v*) dissolved in ethanol–water (20:80, *v*/*v*), hinokitiol at the stated concentration (% *m*/*v*), and/or the commercial products at the stated concentration (% *v*/*v*, as supplied). The specimens were then inoculated with a mixed spore suspension of the three test fungi (*Trichoderma* sp., *Ophiostoma* sp. and *Diplodia seriata*) and incubated at 25 °C and 70–80% relative humidity in the dark inside humidified Petri dishes for 4 weeks.

Three consecutive, independent trials were performed with freshly prepared inocula and treatment solutions; trials 1 and 2 were carried out approximately two months apart. In the first, the fractionated extract was evaluated alone (10%) and in combination (5%) with the commercial anti-sapstain products used by the collaborating sawmill—CP1 (copper-8-quinolinolate, 5.2% *w*/*w*), CP2 (chlorothalonil, 72% *w*/*v*) and CP3 (carbendazim, 50% *w*/*v*)—whose industrial reference mixture contains 4% CP1, 0.5% CP2 and 0.5% CP3 (product basis; Table S7). In the second, hinokitiol (0.025–0.4%) and the extract (0.625–10%) were applied individually to establish their dose–response behavior. In the third, hinokitiol (0.1% or 0.2%) or the extract (5%) was combined with CP1 (1% or 2%) to determine whether chlorothalonil and carbendazim could be omitted and the copper-bearing product reduced; the natural-only combination of hinokitiol (0.4%) with the extract (5%) was assayed under the same conditions. All concentrations refer to the commercial products as supplied (Tables S8–S10).

After incubation, the total fungal growth of the mixed inoculum on each of the ten specimens per treatment was rated visually on a 0–5 scale of surface coverage (0, no detectable growth; 1, growth on ≤20% of the surface; 2, 20–40%; 3, 40–60%; 4, 60–80%; 5, uniform growth over the whole surface), in 0.5-unit steps for intermediate cases. A single rating per specimen was recorded, because the three fungi were co-inoculated and their growth could not be attributed individually on the wood surface. Inhibition was calculated for each treatment as (1 − mean treatment rating/mean untreated-control rating) × 100. Because the scale is ordinal and its categories do not represent equal intervals of colonised area, this percentage is used only as a descriptive index of treatment performance [30]; the specimen-level ratings (Tables S8–S10) were retained as the experimental units for the statistical analysis described in Section 2.9; the same specimens were rated once for the combined growth of the three fungi, so no fungus-specific values exist.

### 2.7. Ecotoxicity Assessment

Acute toxicity bioassays with the freshwater crustacean *Daphnia magna* were commissioned to an external environmental-testing laboratory (ECOMBIO Ltda., Concepción, Chile; report no. 03082022_UDT04) and performed according to the Chilean standards NCh 2083 Of.1999 [31] and NCh 2706 Of.2002 [32], which are harmonised with International Organization for Standardization (ISO) standard 6341 [33,34]. Neonates (<24 h old) were exposed to five concentrations of the selected eucalyptus extract (EBE-S), of hinokitiol and of the copper-8-quinolinolate product CP1, which was tested alone as the synthetic reference; the concentrations were prepared in geometric progression from the undiluted test item (100%) and a dilution-water control (0%) was included. A range-finding test preceded each definitive test. Tests were static without renewal at 20 ± 0.2 °C with a 16 h light/8 h dark photoperiod, using five neonates per 25 mL chamber (20 mL of test solution) and four replicate chambers (20 organisms) per concentration in reconstituted water (hardness 250 mg L^−1^ CaCO_3_, pH 7.8, dissolved oxygen above 80% saturation); control survival above 90% was required for test validity (Table S11). Hinokitiol was tested from a dimethyl sulfoxide (C_2_H_6_OS, DMSO) stock, with the co-solvent limited to avoid solvent toxicity to *D. magna*; because of its low aqueous solubility (1.2 g L^−1^ [35]), the concentration range required to reach 50% mortality could not be attained in true solution, and no LC_50_ is reported for it (Section 3.5). Mortality was recorded after 24 and 48 h and the median lethal concentration (48 h LC_50_) was estimated by the laboratory from the concentration–mortality data; the mortality endpoint reported by the laboratory is retained here, and the term LC_50_ is therefore used throughout. Chamber-level counts and confidence intervals were not included in the laboratory report (Table S12).

### 2.8. Chemical Characterization

#### 2.8.1. Gas Chromatography–Mass Spectrometry (GC-MS)

The non-polar and semi-polar compounds in the fractionated extract were analysed, without derivatisation, on an Agilent 7890A gas chromatograph fitted with a splitless injector and coupled to an Agilent 5975C mass-selective detector (Agilent Technologies, Santa Clara, CA, USA). An HP-5MS column (30 m × 0.25 mm × 0.25 μm; Agilent Technologies) was used for separation, with helium as carrier gas at a constant flow of 1 mL min^−1^. The injector and detector temperatures were set at 250 °C and 280 °C, respectively. The oven temperature was programmed to increase from 60 °C to 300 °C at 10 °C min^−1^. Electron-ionisation (70 eV) spectra were acquired over *m*/*z* 50–550. Compounds were tentatively identified by matching their mass spectra with the NIST 05 library (NIST/EPA/NIH Mass Spectral Library 2005; National Institute of Standards and Technology, Gaithersburg, MD, USA) and with published spectra; a structure was provisionally assigned when the library match exceeded 90%, and for unmatched peaks only the structural skeleton with the highest similarity index is proposed. No authentic standards were analysed and retention indices were not calculated, so all GC-MS assignments, including β-sitosterol and amyrin, should be regarded as tentative. Relative abundances were calculated from the total peak area of the chromatogram.

#### 2.8.2. Ultra-High-Performance Liquid Chromatography–Mass Spectrometry (UHPLC-MS)

The phenolic profile of the fractionated extract was analysed at the Departamento de Análisis Instrumental, Facultad de Farmacia, Universidad de Concepción, on an ultra-high-performance liquid chromatography system coupled to a high-resolution quadrupole time-of-flight mass spectrometer (UHPLC-HR-Q-TOF-MS; Impact II, Bruker Daltonik GmbH, Bremen, Germany). Lyophilized extract (15 mg) was reconstituted in 800 μL of 80% (*v*/*v*) aqueous methanol at 4 °C, homogenised in a mixer mill (MM 400, Retsch GmbH, Haan, Germany; 1 min, 30 Hz), centrifuged (13,000 rpm, 15 min, 4 °C), and 200 μL of the supernatant were transferred to vials with glass inserts for analysis. Separation was performed on a Kinetex C18 column (100 × 3.0 mm, 1.7 μm; Phenomenex, Torrance, CA, USA) fitted with a SecurityGuard Ultra guard cartridge and maintained at 30 °C. The mobile phase consisted of water with 0.1% formic acid (A) and acetonitrile with 0.1% formic acid (B) at a flow rate of 0.4 mL min^−1^; a gradient elution was used (0–2 min, 5% B; 2–20 min, 5–95% B; 20–22 min, 95% B; 22–23 min, 95–5% B; 23–25 min, 5% B) and the injection volume was 2.0 μL. The mass spectrometer was operated in negative electrospray ionisation mode over *m*/*z* 50–2000 (scan cycle 0.2 s) with the following source settings: drying-gas temperature 200 °C, capillary voltage 3.5 kV, end-plate offset 0.5 kV, drying gas (N_2_) 9.0 L min^−1^ and nebuliser pressure 4.0 bar. Sodium formate (10 mM in isopropanol/water, 1:1 *v*/*v*) was injected as internal calibrant at the start of each run and the acquired data were automatically recalibrated in DataAnalysis 4.4 (Bruker; Daltonik GmbH, Bremen, Germany). Features were extracted with MetaboScape 3.0 (T-ReX 3D algorithm), molecular formulas were assigned with SmartFormula3D from the accurate mass and MS/MS fragmentation pattern using a 5 ppm tolerance, and candidate structures were annotated with MetFrag and PubChem and compared with literature data [36]. Identification confidence is reported according to the scheme of Schymanski et al. [37]: level 1, structure confirmed with an authentic reference standard (retention time and MS/MS); level 2, probable structure (molecular formula assigned with |error| ≤ 5 ppm and diagnostic MS/MS product ions matching library or literature data); level 3, tentative candidate (MS/MS product ions consistent with the proposed structure but precursor mass error above 5 ppm); level 4, unequivocal molecular formula only (no MS/MS spectrum). Two compounds, isoquercitrin and quinic acid, were confirmed with analytical standards (Sigma-Aldrich, Merck KGaA, Darmstadt, Germany) at level 1; all other assignments are putative (levels 2–4; Table S6).

### 2.9. Statistical Analysis

Results are presented as mean ± standard deviation (SD; *n* − 1 degrees of freedom). Each of the nine extraction conditions was run in duplicate (*n* = 2). Because only the run means and SDs of the duplicate extractions were archived, the two individual observations of each run were reconstructed as mean ± SD/√2 for the factorial analysis of variance (ANOVA) in Design-Expert^®^; for *n* = 2 this reproduces the run means and the pure-error sum of squares exactly, but it relies on rounded summary values, which is acknowledged as a limitation of the exact p values reported (Table S13). The significance of the factorial-model terms and the test for curvature were assessed against the pure-error variance estimated from the duplicate runs; the normality of the model residuals was examined with the Shapiro–Wilk test (α = 0.05) and residual plots (Table S13); because the duplicate values were reconstructed from the run summaries, a formal homogeneity-of-variance test is not informative and the range of the run standard deviations is reported instead. For the antioxidant assays, the two independent extractions of each run (each measured in technical triplicate and averaged) were the experimental units (*n* = 2 per treatment), and differences between treatment means were analysed by one-way ANOVA followed by Tukey’s HSD test (*p* < 0.05). For the wood-block assays the experimental unit was the individual specimen (*n* = 10 per treatment, one rating of total fungal growth per specimen), and because the response is an ordinal 0–5 score the ratings were analysed within each trial by rank-based non-parametric methods, in keeping with the recommendation that ASTM D4445 ratings be treated as ordinal categories rather than as a continuous response [30]: the Kruskal–Wallis test for the overall treatment effect, the Jonckheere–Terpstra test for monotonic dose–response trends of hinokitiol and of the extract, and Mann–Whitney U tests for a small set of pre-specified within-trial pairwise contrasts, with Holm correction for multiple comparisons (Table S14). Effect sizes are reported as Hodges–Lehmann differences in inhibition (percentage points) with 95% confidence intervals. Mean inhibition percentages are reported as descriptive indices only, and the full distribution of the specimen-level ratings is presented in Section 3.4 rather than means and standard deviations; proportional-odds ordinal models [30] were not fitted because several treatments had identical ratings in all ten specimens. Two comparisons between a natural component applied alone in trial 2 and its combination with 1% CP1 in trial 3 are reported as exploratory, because they are not protected by a common trial. Analyses were performed in Python 3.11 (SciPy 1.x). The absence of a statistically detectable difference is not interpreted as evidence of equivalence. Median lethal concentrations were taken as reported by the external laboratory (Section 2.7).

## 3. Results

### 3.1. Screening of Extraction Conditions Using a 2^3^ Full Factorial Design

The influence of the extraction parameters—temperature (X1: 75–95 °C), ethanol concentration (X2: 40–60% *v*/*v*) and time (X3: 1–3 h)—on the crude extraction yield and antioxidant capacity of *E. globulus* bark extracts was evaluated using a 2^3^ full factorial design with a center point (Tables S1 and S2). Crude yields across the nine runs (STD1–STD9) ranged from 4.12 ± 0.47% (STD8) to 5.94 ± 0.29% (STD2) on an air-dry bark basis, and the antioxidant capacity of the fractionated extracts ranged from 65.4 to 88.2 (DPPH), 80.7 to 106.8 (ABTS) and 122.9 to 141.1 (FRAP) mg GAE g^−1^ (Tables S3 and S4), with no statistically significant differences among treatments in any antioxidant assay (one-way ANOVA and Tukey’s test on the two independent extractions per treatment, *n* = 2, so that the power of this comparison is limited). A first-order polynomial with interaction terms was fitted to the yield data, and the fitted trends are visualized in the response-surface plots presented in Figure 1.

#### 3.1.1. Factorial Model for the Extraction Yield

The first-order model with interaction terms fitted to the yield data, in coded variables (X1 = (Temperature − 85)/10, X2 = (Ethanol% − 50)/10 and X3 = (Time − 2)/1), was (Equation (2)):

Crude Yield (%): Y = 4.98 − 0.29X_1_ + 0.01X_2_ − 0.31X_3_ + 0.05X_1_X_2_ − 0.10X_1_X_3_ − 0.35X_2_X_3_ + 0.15X_1_X_2_X_3_ (2)

The negative coefficients for X_1_ (temperature) and X_3_ (time) indicate that the mildest temperature and the shortest time favored the yield. The main effect of ethanol concentration was negligible, but its interaction with time was the largest term in the model: at 60% *v*/*v* ethanol, prolonging the extraction from 1 to 3 h markedly decreased the yield (5.94% to 4.51% at 75 °C and 5.36% to 4.12% at 95 °C), whereas at 40% *v*/*v* the effect of time was small. The center-point response (5.19 ± 0.34%) was close to the factorial mean (4.98%), indicating no appreciable curvature within the tested ranges. For the antioxidant responses, no coefficient emerged clearly from the experimental variability, consistent with the absence of significant differences among treatments (Table S4); the corresponding surfaces in Figure 1B–D therefore describe trends only. Because the individual duplicate values were reconstructed from the archived run means and SDs (Section 2.9), the exact p values of the model terms should be interpreted with this limitation in mind (Table S13).

#### 3.1.2. Selection of the Most Favourable Extraction Conditions Within the Tested Range

Among the nine experimental runs, treatment STD2 (75 °C, 60% ethanol, 1 h) gave the highest crude yield (5.94 ± 0.29%). Its antioxidant capacity (DPPH 72.7, ABTS 88.7 and FRAP 126.2 mg GAE g^−1^) did not differ significantly from that of the other treatments, whose numerically highest values were found in the 95 °C runs STD8 (DPPH 88.2 and ABTS 106.8 mg GAE g^−1^) and STD6 (FRAP 141.1 mg GAE g^−1^) (Table S4). Because the antioxidant capacity did not discriminate among treatments, the mildest, shortest and highest-yielding condition was selected. As this condition lies at the lower boundary of the temperature domain, it represents the most favorable point within the explored range rather than a true interior optimum, and temperatures below 75 °C may merit future evaluation. The extract obtained under these conditions (henceforth referred to as the selected extract, EBE-S) was used for all subsequent characterization and bioactivity testing.

### 3.2. Extract Fractionation and Purification

The crude extracts from all nine treatments underwent methanol precipitation fractionation to remove interfering carbohydrates and concentrate bioactive phenolic and terpenoid compounds. Cold methanol treatment (4:1 *v*/*v*) selectively precipitated polysaccharides while maintaining phenolic and terpenoid compounds in solution. The fractionation efficiency varied considerably among treatments, with removed carbohydrates accounting for 6–25% of the crude extract mass and the total phenolic content of the fractionated extracts ranging from 560 to 790 mg GAE g^−1^ (Table 1).

Treatment STD8 showed the highest total phenolic content (790 mg GAE g^−1^)—consistent with its numerically highest DPPH and ABTS values (Table S4)—despite having the lowest carbohydrate removal rate (7%), suggesting that this treatment initially produced a crude extract with lower carbohydrate content. In contrast, STD6 demonstrated the highest carbohydrate removal efficiency (25%), reducing residual sugars to only 8%. Treatment STD2, selected in Section 3.1.2, showed moderate fractionation performance with 16% sugar removal and a total phenolic content of 660 mg GAE g^−1^, indicating a balanced composition suitable for antifungal applications.

Whether the fractionation step concentrated the bioactive compounds cannot be established from these data, because a paired comparison of crude and fractionated material from the same extraction was not performed. The residual carbohydrate content (7–23%) suggests that complete removal was not achieved with the methanol precipitation method alone, which is consistent with the presence of low molecular weight sugars and glycosylated phenolic compounds that remain soluble in methanol [19,38]. This incomplete separation may actually be beneficial, as glycosylated flavonoids have been reported to exhibit biological activity and may contribute to the overall antifungal efficacy of the extracts [39].

### 3.3. Chemical Characterization of the Selected Extract (EBE-S)

#### 3.3.1. GC-MS Analysis of Non-Polar and Semi-Polar Compounds

The GC-MS analysis of the fractionated EBE-S tentatively identified eleven compounds, accounting for 75.73% of the total chromatogram area (Table 2). The chemical profile was dominated by sterols and triterpenes: β-sitosterol, a well-known phytosterol with reported antimicrobial properties, was the most abundant compound (37.29% of the relative abundance), followed by amyrin (20.78%), a pentacyclic triterpene also known for its antifungal and antioxidant activities. Other constituents included the sesquiterpenes γ-gurjunene (5.58%) and aromadendrene (2.50%), the sesquiterpenols globulol (1.23%) and α-eudesmol (0.58%), the dihydroisocoumarin mellein (1.65%), methyl oleate (1.13%) and 3,4,5-trimethoxyphenol (0.59%). Two minor compounds, 1,3-di-tert-butylbenzene (3.63%) and 2,5-di-tert-butylphenol (0.77%), are common synthetic antioxidants or plasticizer-related compounds and most probably represent contaminants from the extraction process, solvents or labware rather than genuine bark constituents; they were therefore excluded from the mechanistic discussion.

#### 3.3.2. UHPLC-MS Analysis of Phenolic Compounds

The phenolic profile of the EBE-S was further detailed by UHPLC-MS, which allowed the annotation of fourteen compounds and one additional isomer, two of which (isoquercitrin and quinic acid) were confirmed with authentic standards; six annotations whose precursor mass error exceeded 5 ppm are reported as tentative (level 3) (Table 3). The profile was dominated by ellagic acid derivatives (ellagic acid glucoside, 3-methylellagic acid 8-(2-acetylrhamnoside), 2,3-di-O-methylellagic acid and 2,3,8-tri-O-methylellagic acid), flavonoid glycosides (isoquercitrin, kaempferol-7-O-glucuronide, naringenin-7-O-glucoside and 6-methoxytaxifolin), galloyl derivatives (1-galloyl-β-D-glucose and a trimethoxyphenyl-galloyl-glucopyranoside), quinic acid and the acetophenone glycoside erythroxyloside B, together with the triterpenoid acids poricoic acid K and acacic acid. The presence of these diverse structures, including organic acids, flavonoids and hydrolysable-tannin derivatives, is consistent with the extract’s observed antioxidant activity. The remaining assignments are based on accurate mass and MS/MS fragmentation patterns only (molecular formulas, calculated and observed *m*/*z*, mass errors, diagnostic product ions and identification levels are listed in Table S6); compounds identified on this basis alone—including poricoic acid K (*m*/*z* 485.3272), a triterpenoid acid reported mainly from the fungus *Wolfiporia cocos*—are tentative and require confirmation with authentic standards.

#### 3.3.3. Mechanistic Hypotheses Derived from the Chemical Characterization

Following the GC-MS and UHPLC-MS analyses, the literature on the detected constituents was reviewed to identify the mechanisms that could underlie the antifungal activity observed against the wood-staining fungi, together with the decay-oriented mechanisms reported for the same compound classes. The outcome is summarised in Table 4.

For the detected constituents and their compound classes, the activities and mechanisms reported in the literature comprise metal chelation by galloyl and ellagic acid derivatives and flavonol glycosides, inhibition of cellulolytic enzymes, membrane perturbation by β-sitosterol and amyrin, and the susceptibility of flavonoids such as naringenin and kaempferol to fungal laccases and cytochrome P450 monooxygenases [15,40,41,42,43]. These mechanisms are hypotheses derived from the literature; none was measured in this study, and they are considered in Section 4 only in so far as they bear on the moderate standalone efficacy of the extract and on the rationale for hybrid formulations.

**Table 4:** Constituents detected in the extract and their compound classes, with antifungal activities and mechanisms reported for *Eucalyptus globulus* bark. Free gallic acid and condensed tannins, which are reported for *E. globulus* bark but were not detected in this extract, are not listed; the corresponding literature is cited in refs [15,40,44,45,46].

Compound	Activity Against Wood Fungi	Metal Chelation & Enzyme Inhibition	Membrane & Ion Homeostasis Effects	Reference
Ellagic acid	Inhibits wood-decay fungi. More effective against brown-rot than white-rot fungi. Documented for wood protection applications.	Metal chelator (Fe, Cu). Inhibits cellobiose dehydrogenase (CBH1), β-glucosidase (BGL1), and endoglucanase (EG2). Interferes with ergosterol biosynthesis. Binds to fungal proteins.	Inhibits fungal cell wall synthesis. Disrupts membrane integrity.	[15,41,47,48]
Naringenin-7-O-glucoside	Antifungal against *Diplodia* spp. (wood-staining fungus). Effective against phytopathogenic fungi that colonize wood. White-rot fungi (*Phanerochaete chrysosporium*) can oxidize naringenin via CytP450 enzymes.	Moderate Fe/Cu chelator. Substrate for fungal CytP450 enzymes (detoxification pathway). Glycoside form enhances water solubility and penetration into wood.	Disrupts fungal membrane integrity. Glycosides may be hydrolyzed to active aglycones in wood matrix.	[15,42,43]
Kaempferol-7-O-glucuronide	Inhibits wood-degrading fungi. Effective against phytopathogenic fungi. Glycosides show enhanced bioavailability in wood matrix. Susceptible to laccase-mediated degradation by white-rot fungi.	Strong Fe/Cu chelator (3-hydroxyl-4-keto structure). Multi-target: inhibits laccases, cellulases, and glucosidases. Reduces metal availability for fungal enzymes. Oligomeric flavonoids exert more inhibition than monomers.	Membrane disruption. ROS scavenging. Perturbation of ionic homeostasis.	[15,43,49,50]
Quinic acid	Antibiofilm agent (relevant for fungal colonization of wood surfaces). Enhances efficacy of other biocides. May prevent initial fungal attachment to wood.	Disrupts biofilm formation; affects fungal cell viability. May interfere with fungal quorum sensing. Enhances penetration of other antifungal compounds.	Alters cell wall biosynthesis. Affects membrane permeability.	[51,52]
β-Sitosterol	Membrane-active compound. Alters fungal membrane fluidity and permeability. Effective against wood-staining and decay fungi.	Competes with ergosterol in fungal membranes. May interfere with sterol biosynthesis pathways.	Disrupts membrane lipid bilayers. Alters fluidity and permeability of cellular membranes. Affects membrane-bound enzyme activities.	[15,41]
Amyrin	Membrane-active compound. Antimicrobial activity against fungi and bacteria.	May interfere with fungal enzyme systems. Lipophilic nature allows interaction with membrane-bound proteins.	Disrupts membrane integrity. Alters membrane permeability. Affects ion transport across membranes.	[41]

### 3.4. Antifungal Efficacy Assessment

#### 3.4.1. In Vitro Antifungal Screening

Initial screening of the nine reactor extracts (STD1–STD9) using disc- and agar-diffusion methods showed limited and selective antifungal activity. At 50–100 mg mL^−1^ none of the extracts produced a fungistatic or fungicidal effect against any of the test fungi. When the agar-diffusion assay was repeated at higher concentrations (500 and 1000 mg mL^−1^), a concentration-dependent fungistatic effect was observed only against *Diplodia seriata*, with growth inhibition of approximately 30% relative to the control plates; the strongest responses corresponded to extracts STD2 and STD4. Both positive controls, CP4 and the CP1–CP3 industrial mixture (Section 2.5), produced complete inhibition (100%) of fungal growth under the same conditions. This selective activity suggests that the extract’s components are more effective against specific fungal species and that the extract may not function as a broad-spectrum fungicide when used alone. This limited standalone activity is consistent with reports for other bio-based wood preservatives: Barbero-López et al. [53] found that bark and wood extracts applied alone gave only partial protection against sapstain and mould fungi and were markedly less effective than a commercial reference unless combined with additional actives, a pattern that mirrors the behavior observed here.

#### 3.4.2. Wood Preservation Efficacy and Hybrid Formulations

The efficacy of the extract and its formulations in protecting wood specimens was evaluated according to the ASTM D4445-10 standard. The results, summarized in Figure 2, highlight the limitations of the natural products when used alone and the performance of the natural combination and of the hybrid formulations with commercial products.

Applied individually, the natural components confirmed the in vitro picture. The extract did not exceed 40% inhibition at any concentration tested: 9% at 0.625–1.25%, 27% at 2.5%, 39% at 5% and 40% at 10% in the dose–response trial (Table S9), and 33% at 10% in the first trial (Table S8). Hinokitiol, in contrast, was strongly dose-dependent, rising from 64% inhibition at 0.025% to 73% at 0.05%, 82% at 0.1% and 91% at 0.2%, and reaching complete inhibition (100%) at 0.4%; across the five tested levels this monotonic dose–response was supported by the Jonckheere–Terpstra trend test on the specimen-level ratings (*n* = 50; JT = 920, z = 7.41, *p* < 0.001; Kruskal–Wallis H = 41.8, df = 4, *p* < 0.001). For the extract, inhibition likewise increased with concentration (JT = 892, z = 6.96, *p* < 0.001; Kruskal–Wallis H = 43.7, df = 4, *p* < 0.001) but plateaued below 40% (Table S9). The lower value obtained for the 10% extract in trial 1 (33%) than in trial 2 (40%) is attributed to between-trial variability: the two trials were carried out about two months apart with independently prepared inocula and treatment solutions and, although the incubation parameters were controlled, differences of this magnitude are expected for a visual 0–5 rating of independent runs.

The natural-only combination of hinokitiol (0.4%) with the extract (5%) gave 100% inhibition in trial 2 and 95% in trial 3, a level similar to that of the fully synthetic reference. Because hinokitiol alone already reached 100% at 0.4%, the addition of the extract produced no detectable change (Mann–Whitney U = 35, *n* = 10 + 10, Holm-adjusted *p* = 0.39; Hodges–Lehmann difference 0 percentage points, 95% CI −10 to 0; Table S14), so its incremental contribution at this dose cannot be resolved.

The hybrid formulations with commercial products showed similarly high observed inhibition while reducing the synthetic load. In the first trial, every mixture of the extract (5%) containing at least 2% of the copper-8-quinolinolate product CP1 reached 100% inhibition, irrespective of whether chlorothalonil (CP2) or carbendazim (CP3) was present, whereas the extract with 0.25% CP2 alone reached only 47% (Table S8); the half-dose synthetic mixture (2% CP1, 0.25% CP2 and 0.25% CP3) gave 93% in the same trial, whereas the full industrial mixture gave 100% (Table S8). Without the copper product, the extract (5%) with 0.25% CP3 reached 87% and with 0.25% CP2 plus 0.25% CP3 93%, both detectably higher than with 0.25% CP2 alone (Holm-adjusted *p* < 0.001; Hodges–Lehmann differences +40 and +50 points; Table S14, contrasts T1–T2). In the third trial, formulations in which chlorothalonil and carbendazim were omitted entirely—hinokitiol (0.1–0.2%) or the extract (5%) combined with 1–2% CP1—maintained 90–100% inhibition (Kruskal–Wallis across the eleven treatments of trial 3, H = 60.5, df = 10, *p* < 0.001), a level similar to that of the synthetic reference mixture included in the same trial (100%), while halving the CP1 dose relative to the 4% used industrially (Table S10). On an active-ingredient basis (all products dosed by volume; CP1 density 1.05 g cm^−3^ and 5.2% *w*/*w* copper-8-quinolinolate; CP2 72% *w*/*v* chlorothalonil; CP3 50% *w*/*v* carbendazim), the industrial reference contains 0.218 g of copper-8-quinolinolate, 0.360 g of chlorothalonil and 0.250 g of carbendazim per 100 mL (0.828 g of synthetic actives), whereas the hybrid formulations with 2% and 1% CP1 contain 0.109 and 0.055 g per 100 mL, respectively, i.e., reductions of 87% and 93% in synthetic active ingredients (Table S7). It should be noted that CP1 at 2% alone also achieved 100% in this trial, so the specific contribution of the natural components at that dose cannot be isolated; at 1% CP1, the combinations with the extract or hinokitiol still retained 90–93% inhibition. Within trial 3, the extract 5% + 1% CP1 (93%; Holm-adjusted *p* = 0.21) and hinokitiol 0.1% + 1% CP1 (93%; adjusted *p* = 0.39) did not differ detectably from the synthetic reference, whereas hinokitiol 0.2% + 1% CP1 (90%) was detectably lower (U = 20, adjusted *p* = 0.040; Hodges–Lehmann difference −10 points, 95% CI −20 to 0); at 2% CP1 none of the three combinations differed detectably from the copper product alone (adjusted *p* = 0.50, 0.74 and 1.0 for the extract, hinokitiol 0.2% and hinokitiol 0.1%, respectively; Hodges–Lehmann differences of 0 points), consistent with the copper product being sufficient for complete control at that dose (Table S14). Comparing across trials, which is exploratory because the observations are not protected by a common run, adding 1% CP1 raised the observed efficacy of the extract from 39% (trial 2) to 93% (trial 3) (U = 100, *p* < 0.001; Hodges–Lehmann difference +60 points, 95% CI 50 to 60), whereas for hinokitiol at 0.2% the same addition produced no detectable change (91% vs. 90%; U = 47, *p* = 0.84; difference 0 points, 95% CI −10 to 10). All within-trial contrasts were pre-specified and Holm-corrected (Section 2.9), and the absence of a detectable difference is not interpreted as equivalence.

### 3.5. Acute Aquatic Toxicity of the Extract and the Copper-8-quinolinolate Reference

The acute toxicity of the EBE-S, of hinokitiol and of the copper-8-quinolinolate product CP1 was evaluated using *Daphnia magna* to assess their environmental impact potential (Figure 3). The eucalyptus bark extract itself showed low acute toxicity, with a 48 h LC_50_ of 27,500 ppm. In contrast, CP1, the copper-8-quinolinolate product of the industrial reference mixture, was markedly more toxic to *D. magna*, with a 48 h LC_50_ of 38.71 ppm (product basis; approximately 2.0 ppm of copper-8-quinolinolate according to the declared 5.2% *w*/*w* content). On a per-mass basis, the extract was therefore approximately 710 times less acutely toxic than CP1; because chlorothalonil and carbendazim were not tested, this ratio refers to the copper product alone and not to the complete industrial mixture. For hinokitiol, no valid LC_50_ could be derived: its aqueous solubility (≈1.2 g L^−1^ [35]) lies far below the concentration range that would have been required to produce 50% mortality, so the nominal concentrations tested did not correspond to dissolved exposure concentrations and the result fell outside the workable scale of the assay; the value is therefore not reported. In addition, as an iron-chelating tropolone, hinokitiol is prone to metal complexation and precipitation under the test conditions (hardness 250 mg L^−1^ CaCO_3_, pH 7.8). Regulatory categorisation has classed hinokitiol as non-persistent, non-bioaccumulative and not inherently toxic to aquatic organisms [54], whereas its structural analogue tropolone is classified as very toxic to aquatic life; a dedicated determination under OECD 202/ISO 6341 with analytical verification of the exposure concentration is required before any aquatic-safety statement can be made for it. However, because the extract was applied to wood at 5% (≈50,000 ppm)—a nominal concentration above its own 48 h LC_50_—this ratio reflects the intrinsic hazard of the material rather than the realised aquatic risk of the treated wood, which depends on the fraction that leaches into water; quantifying leaching and fixation is therefore an essential prerequisite before any environmental-safety advantage can be asserted.

## 4. Discussion

The 2^3^ factorial screening of the extraction conditions showed that the lowest tested temperature of 75 °C, ethanol 60% and a time of 1 h (STD2) combined the highest crude yield (5.94%) with the mildest processing conditions. Antioxidant capacity, in contrast, did not differ significantly among treatments and was numerically highest in the 95 °C runs, so the milder condition was preferred on yield grounds. The yield decline observed at 95 °C may reflect thermal degradation of major bioactive compounds, as is commonly reported for complex mixtures of natural products, although chemical stability and degradation products were not measured here [9,15].

A key issue that emerged from our analysis is the apparent co-extraction of carbohydrates, which still amounted to 7–23% even after methanol precipitation. This value, however, must be interpreted with caution. The phenol-sulfuric acid method relies on the acid hydrolysis of all glycosidic bonds and therefore overestimates the content of free monosaccharides, since it also quantifies the sugar moieties covalently bound to the glycosylated phenolics identified by UHPLC-MS (naringenin-7-O-glucoside, kaempferol-7-O-glucuronide and 1-galloyl-β-D-glucose). Rather than representing a readily available nutrient source for fungal growth, a substantial fraction of this signal corresponds to structurally bound sugars; moreover, glycosylation improves the water solubility and penetration of these bulky phenolics into the hydrophilic wood matrix, and the conjugates can act as a controlled-release reservoir from which fungal β-glucosidases liberate the active aglycones in situ [39,41,55]. However, this also provides a very apparent opportunity. A more specific and efficient removal of these carbohydrates (aside from enzymatic hydrolysis) or advanced type of chromatography could help greatly in the concentration of the bioactive phenolics and terpenoids, in order to further enhance the intrinsic antifungal potency of the extract. Non-tannin constituents, and free sugars in particular, are known to impair the performance of bark-derived wood preservatives: they increase leaching and can serve as a readily available carbon source for colonising fungi [56,57,58]. The carbohydrate signal quantified here (Table 1) represents total carbohydrates, including the sugar moieties of glycosylated phenolics, and should not be equated with free fermentable sugars [36]. A selective removal of the fermentable fraction by microbial fermentation, for instance with *Saccharomyces cerevisiae*, which tolerates tannin-rich media and has been used to ferment bark-containing liquors [59,60,61], could therefore be an inexpensive alternative to chromatographic clean-up; whether this would improve the anti-sapstain performance of the extract is a hypothesis that remains to be tested. On this basis, the moderate standalone efficacy observed on wood (≤40%) may represent a baseline that further purification could improve.

The combination of lipophilic terpenoids (identified by GC-MS) and polar phenolics (identified by UHPLC-MS) may act on fungal cells on several fronts, as summarised in Table 4; the mechanisms discussed below are inferred from the literature and were not measured in this study.

Before discussing these mechanisms, it is important to distinguish the two functional groups of wood-inhabiting fungi they target, because the test organisms used in this study (*Ophiostoma* sp., *Diplodia seriata* and *Trichoderma* sp.) are wood-staining (sapstain) fungi rather than true wood-decay fungi. Sapstain fungi do not extensively depolymerise the structural cellulose and lignin of the cell wall; instead, they colonise the sapwood through rays and resin ducts and grow by assimilating the readily available carbon and nitrogen nutrients stored in the parenchyma cells, producing discoloration mainly through melanised hyphae [62,63]. Among the test fungi, *D. seriata* (Botryosphaeriaceae) is a sapwood-colonising ascomycete associated with discoloration and canker diseases of woody hosts; it secretes cellulolytic and hemicellulolytic enzymes as well as laccase and Mn-dependent peroxidase and possesses a lignocellulolytic gene repertoire [64,65], so it can modify cell-wall components to a greater extent than the ophiostomatoid stain fungi. It is, however, distinct from *Diplodia sapinea* (syn. *Sphaeropsis sapinea*), the species classically associated with blue stain of Pinus radiata [66], and the diagnostic features of soft rot (type I cavities or type II erosion, mass loss and loss of strength) have not been reported for it [67,68]; it is therefore treated here as a staining fungus with cell-wall-modifying capacity rather than as a decay fungus. Consequently, for the organisms tested here the most directly operative mechanisms of the extract are the deprivation of essential metal ions and nutrients and the disruption of the fungal membrane, rather than the inhibition of lignocellulolytic enzymes. Nevertheless, an effective wood-protection system should also remain active against the wood-decay fungi (brown- and white-rot) that frequently colonise the same substrate once staining has facilitated moisture uptake; the broader enzymatic and Fenton-related mechanisms summarised in Table 4 are therefore relevant as additional, decay-oriented modes of action of the same chemical constituents, although they were not tested here [15,41].

Despite this chemical diversity, the efficacy of the extract was moderate by itself. This may be explained by the detoxification capacity of wood-inhabiting fungi: staining fungi, like the lignolytic wood-decay fungi, possess oxidative and conjugating enzyme systems (including laccase-like phenoloxidases and cytochrome P450 monooxygenases) that can transform and inactivate phenolic compounds. As detailed by Valette et al. [15] and indicated in Table 4, fungi can counteract phenolic compounds through extracellular oxidases such as laccases and intracellular detoxification systems such as cytochrome P450 monooxygenases (CytP450) [15]. These enzymes can transform many of the extract’s primary components; the flavonoids naringenin and kaempferol, for example, are known substrates of these fungal enzymes [42].

This fungal counter-defence offers a rationale for why hybrid formulations may be required to achieve robust and long-lasting protection. The eucalyptus extract, although chemically diverse, can be slowly degraded by these enzymatic systems, whereas hinokitiol, a tropolone, is considerably more resistant to enzymatic degradation. Consistent with this rationale, the combination of the extract (5%) with hinokitiol (0.4%) reached 95–100% inhibition on wood. Because hinokitiol alone at 0.4% also achieved complete inhibition, the contribution of the extract at that dose cannot be isolated from the present data, and the study did not quantify the interaction with a formal index (e.g., the fractional inhibitory concentration index, FICI, or Abbott’s method). The combined treatments are therefore described as effective rather than synergistic, and the distinction between additivity and true synergy remains to be established with a fixed-ratio design at sub-maximal hinokitiol doses (0.1–0.2%).

Our results may have important implications for a new generation of sustainable wood preservatives. The strict requirements of the Biocidal Products Regulation (BPR) and the restriction of toxic chemicals such as CCA have driven the market demand for natural and safer wood preservatives [4,5]. The search for a single natural compound that could substitute synthetics, however, has been largely unsuccessful, because of the natural resistance of wood-destroying fungi [38,53].

Our findings highlight the value of hybrid systems that combine existing commercial products with natural components. Formulations without chlorothalonil or carbendazim—whether fully natural (extract 5% + hinokitiol 0.4%; 95–100%) or based on a halved or quartered dose of the copper-8-quinolinolate product (97–100% and 90–93%, respectively)—retained high efficacy, a promising step towards preservatives with a reduced synthetic load. The lower acute toxicity measured here (approximately 710-fold on a per-mass basis) applies to the bark extract relative to the copper-8-quinolinolate product only; no valid toxicity value could be established for hinokitiol and the final formulations were not tested, so any environmental advantage is at present confined to the extract and the copper-hybrid route and remains to be verified for the hinokitiol-based combination. Because the current cost of hinokitiol at the fully natural dose is a barrier to industrial scale-up, the copper-hybrid route offers a pragmatic intermediate.

The resilience of wood-staining fungi, which often grow as protective biofilms, has been argued to require such a multi-component approach rather than a single active ingredient [62,69]. An effective formulation must combine several compounds with complementary mechanisms of action, as in our hybrid system, which integrates metal-chelating phenolics, membrane-disrupting terpenoids and a potent, stable biocide such as hinokitiol or copper-8-quinolinolate. Such a multi-target combination may provide a defence that is more difficult for the colonising fungi to overcome, a hypothesis that the present data do not test directly.

This study has limitations that delimit its conclusions. Efficacy was assessed in a four-week laboratory assay; long-term performance, leaching resistance, photostability and formulation shelf-life were not evaluated. The acute aquatic toxicity of the final formulations was not measured, and the *Daphnia magna* data were obtained by an external laboratory that reported point estimates without confidence intervals. The test organisms were sapstain and surface-mold fungi, and activity against wood-decay (brown- and white-rot) basidiomycetes—for which several of the proposed mechanisms (Fenton chemistry, cellulolytic-enzyme inhibition) are most relevant—was inferred from the literature rather than measured. Penetration of the extract into pine sapwood was not quantified, and the mechanisms of action discussed here were drawn from the literature and not measured experimentally. The trial design also lacked a 1% CP1-only control, so the incremental contribution of the natural components at the lowest copper dose could not be isolated. These aspects, together with formal synergy quantification (FICI), a dedicated determination of the acute aquatic toxicity of hinokitiol under OECD 202/ISO 6341 with analytical verification, standardized decay tests (European standard EN 113 [70] and American Wood Protection Association standard AWPA E10 [71]), leaching tests (EN 84 [72]) and field exposure, should be addressed in future work.

## 5. Conclusions

This study indicates that, although *Eucalyptus globulus* bark extract exhibits limited standalone efficacy (≤40% inhibition), it is a candidate bioactive component of reduced-synthetic (hybrid) wood-preservative systems. A 2^3^ full factorial design with a center point identified the most favourable extraction conditions within the tested ranges (75 °C, 60% *v*/*v* ethanol, 1 h), yielding a chemically diverse extract characterized by lipophilic triterpenoids (β-sitosterol, amyrin) and polar phenolics (ellagic acid derivatives, galloyl esters and flavonoid glycosides).

The main finding of this work is that, in four-week laboratory wood-block assays, fungal staining was controlled by formulations with a substantially reduced load of conventional synthetic biocides. The central lesson is that the hybrid route is viable whereas the extract alone is not: on its own the extract provides only a partial, species-selective barrier, but when paired with hinokitiol or with a halved dose of the copper-8-quinolinolate product it showed similarly high observed inhibition to the conventional industrial mixture while allowing chlorothalonil and carbendazim to be omitted; because the copper product alone was also fully effective at 2%, the incremental contribution of the natural components at that dose could not be isolated. Because the cost of hinokitiol at the fully natural dose is a barrier to scale-up, the copper-hybrid formulation is the pragmatic near-term option. The lower acute toxicity of the extract relative to the copper-8-quinolinolate product was confirmed in *Daphnia magna* bioassays.

These results indicate that reducing synthetic-biocide use in wood protection may be achievable through multi-component natural–synthetic formulations rather than single-active replacement. These hybrid formulations may exploit multi-target mechanisms (metal chelation, enzyme inhibition, membrane disruption) inferred from the literature; in the laboratory assays they allowed chlorothalonil and carbendazim to be omitted and the copper-8-quinolinolate product to be halved, an overall reduction of about 87% in synthetic active ingredients, while retaining similarly high observed efficacy. Their environmental performance, leaching behaviour and durability remain to be confirmed before they can be regarded as replacements for the conventional mixture. Future efforts should focus on quantifying leaching and fixation of the active constituents, confirming efficacy against wood-decay basidiomycetes in standardized decay tests, establishing by means of a fixed-ratio design whether the combined effect is additive or truly synergistic, and assessing field durability, photostability and formulation shelf-life.

## Figures and Tables

**Figure 1:**
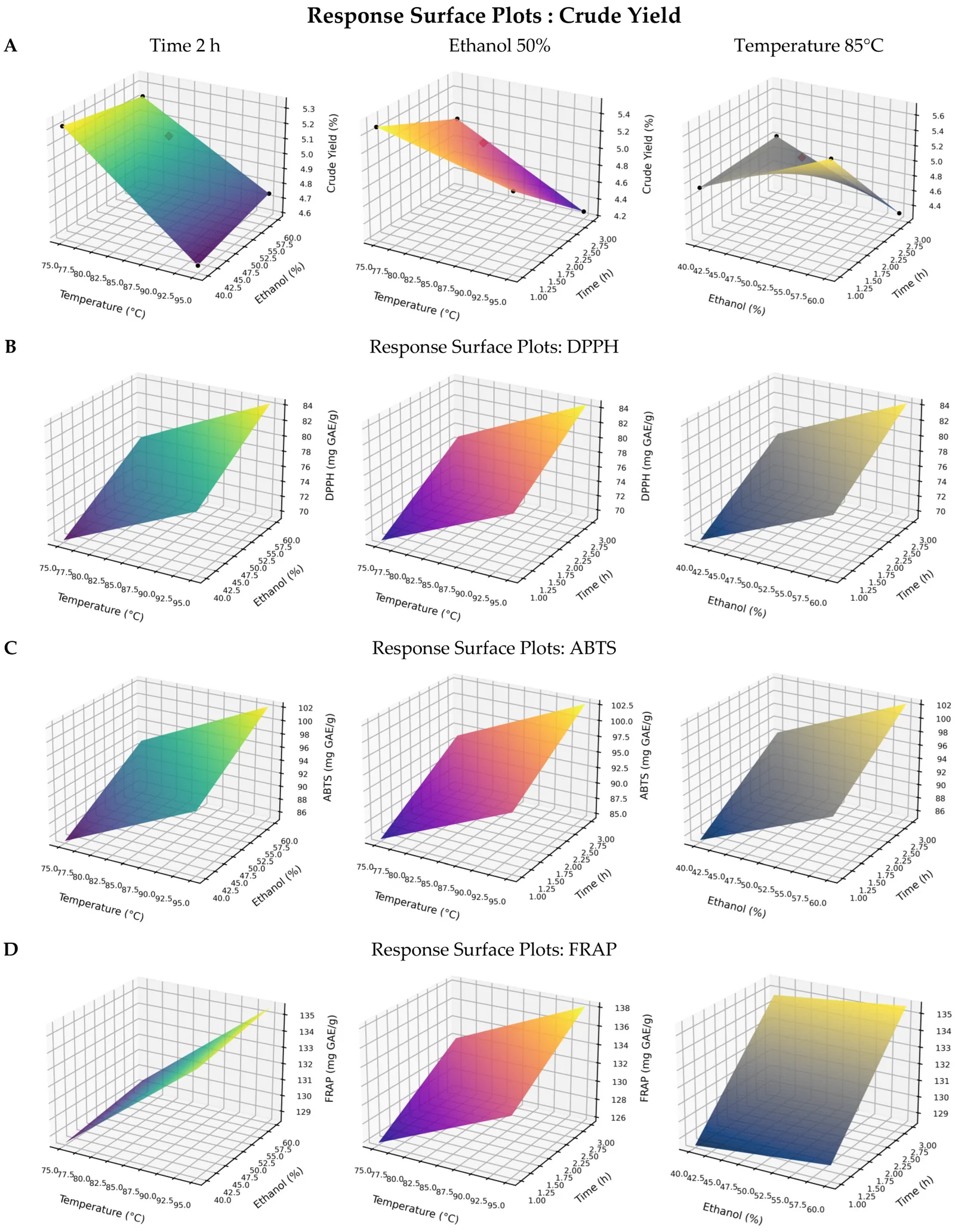
Response surface plots illustrate the effects of temperature, ethanol concentration, and time on (**A**) crude yield and antioxidant activities: (**B**) DPPH, (**C**) ABTS, and (**D**) FRAP of *E. globulus* bark extracts. The surfaces were generated from the first-order factorial models with interaction terms fitted to the experimental data (2^3^ design with center point). For each response, the three surfaces show the fitted model over each factor pair, holding the third factor at its center level (85 °C, 50% *v*/*v* ethanol or 2 h); symbols are observed run means averaged over the held factor, and the center run (STD5) is shown at its observed value. Surface shading maps the fitted response value (darker to lighter, lower to higher) and carries no additional information.

**Figure 2:**
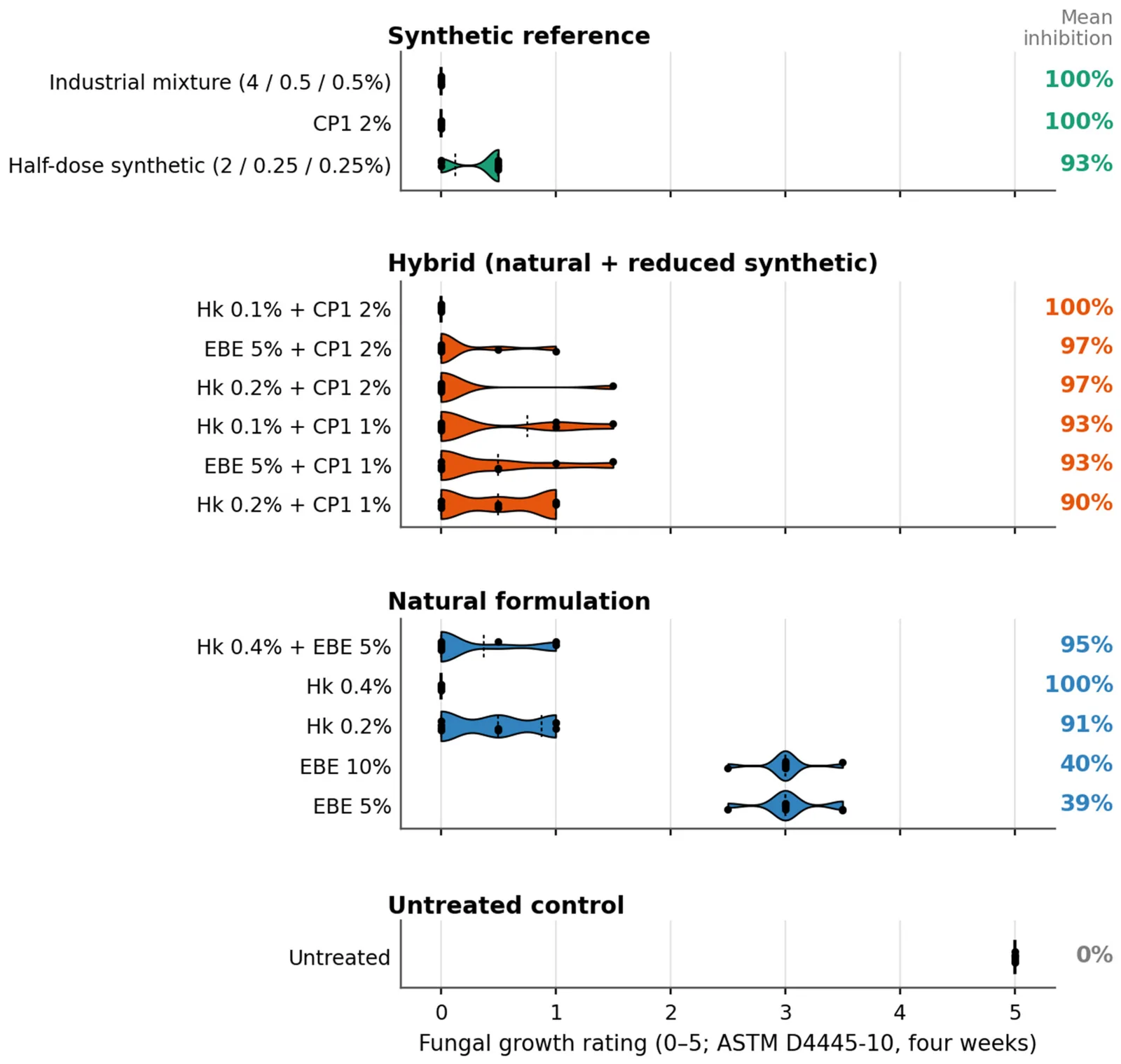
Fungal growth ratings (0–5 scale; ASTM D4445-10, four-week incubation; mixed inoculum of the three test fungi) on wood specimens treated with the *E. globulus* bark extract, hinokitiol (Hk), their natural-only combination, and formulations with the commercial copper-8-quinolinolate product CP1, grouped by category. The extract alone did not exceed 40% inhibition; hinokitiol reached complete inhibition at 0.4%; the natural combination (0.4% hinokitiol + 5% extract) reached 95%; and formulations of either component with 1–2% CP1 maintained 90–100% inhibition, allowing chlorothalonil and carbendazim to be omitted and the copper product halved relative to the sawmill reference mixture. Violin plots show the kernel density of the ten specimen-level ratings of each treatment (one rating of total fungal growth per specimen; density truncated at the observed range; dashed lines, quartiles), with the individual specimens overlaid; a vertical line denotes treatments in which all ten ratings were identical. Ratings follow the ASTM D4445-10 visual scale of surface coverage by fungal growth: 0, no detectable growth; 1, ≤20% of the surface; 2, 20–40%; 3, 40–60%; 4, 60–80%; 5, uniform growth over the whole surface. The mean inhibition (%) relative to the untreated control (rating 5 in all specimens) is given on the right as a descriptive index. The corresponding statistics are given in Table S14.

**Figure 3:**
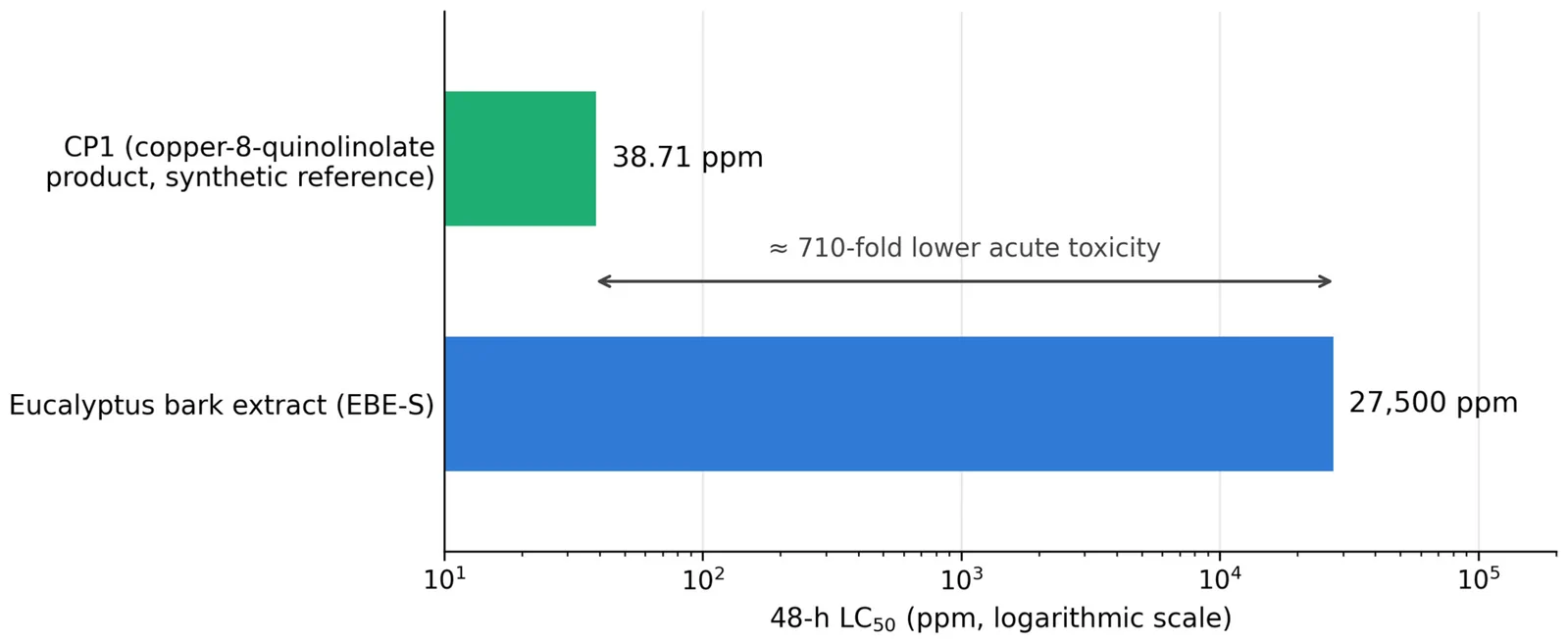
Acute toxicity (48 h median lethal concentration, LC_50_, in ppm) of the *Eucalyptus globulus* bark extract and the copper-8-quinolinolate product CP1 (synthetic reference) against *Daphnia magna*, plotted on a logarithmic scale (higher LC_50_ = lower toxicity). The extract (27,500 ppm) showed low acute toxicity, whereas CP1 (38.71 ppm) was approximately 710-fold more toxic on a product-mass basis. Values are the 48 h LC_50_ point estimates reported by the external laboratory; confidence intervals were not provided (Table S12).

**Table 1:** Fractionation efficiency and composition of eucalyptus bark extracts after methanol precipitation.

Treatment	Sugars Removed (%)	Residual Sugars (%)	Total Phenolic Content (mg GAE g^−1^)
STD 1	24	15	580
STD 2	16	13	660
STD 3	21	18	560
STD 4	9	13	730
STD 5	16	16	630
STD 6	25	8	620
STD 7	12	16	670
STD 8	7	7	790
STD 9	6	23	660
Mean	15.1 ± 7.1	14.3 ± 5.0	656 ± 75

Note: Sugars removed and residual sugars were quantified by the phenol-sulfuric acid method and are expressed as glucose equivalents (% *w*/*w* of the crude extract dry weight). Total phenolic content was determined by the Folin–Ciocalteu method and is expressed as mg gallic acid equivalents per g of fractionated extract (mg GAE g^−1^); as an assay response it is not a gravimetric mass fraction and no mass balance among the columns is implied.

**Table 2:** Compounds tentatively identified (NIST library match; no authentic standards) in the fractionated eucalyptus bark extract by GC-MS analysis.

Compound	Relative Abundance (%)	Class	Reported Biological Activity
β-Sitosterol	37.29	Sterol	Antimicrobial, Anti-inflammatory
Amyrin	20.78	Triterpene	Antifungal, Antioxidant
γ-Gurjunene	5.58	Sesquiterpene	Antifungal
1,3-Di-tert-butylbenzene	3.63	Alkylbenzene	Probable process contaminant
Aromadendrene	2.50	Sesquiterpene	Antimicrobial
Mellein	1.65	Dihydroisocoumarin	Antifungal
Globulol	1.23	Sesquiterpenol	Antifungal, Antimicrobial
Methyl oleate	1.13	Fatty acid methyl ester	—
2,5-Di-tert-butylphenol	0.77	Phenolic	Antioxidant; probable contaminant
3,4,5-Trimethoxyphenol	0.59	Phenolic	Antifungal
α-Eudesmol	0.58	Sesquiterpenol	Antimicrobial
Total identified	75.73		
Unidentified	24.27	Unknown	Unknown

**Table 3:** Compounds annotated in the fractionated eucalyptus bark extract by UHPLC-HR-Q-TOF-MS in negative ionization mode, with molecular formula, observed [M − H]^−^ *m*/*z*, mass error relative to the calculated value and identification level [37] (full data in Table S6).

No.	RT (min)	Putative Compound	Formula	Obs. [M − H]^−^ (*m*/*z*)	Error (ppm)	Level ^a^
1	1.70	Quinic acid	C_7_H_12_O_6_	191.0557	−2.2	1
2	3.56	1-Galloyl-β-D-glucose	C_13_H_16_O_10_	331.0687	4.9	2
3	7.32	Ellagic acid glucoside	C_20_H_16_O_13_	463.0521	0.6	2
4	8.53	Trimethoxyphenyl-galloyl-glucopyranoside	C_22_H_26_O_13_	497.1267	−6.8	3
5	8.70	Erythroxyloside B	C_21_H_30_O_13_	489.1580	−6.9	3
6	9.36	Isoquercitrin	C_21_H_20_O_12_	463.0848	−7.3	1
7	10.50	Kaempferol 7-O-glucuronide	C_21_H_18_O_12_	461.1707 ^b^	—	3
8	11.20	Naringenin 7-O-glucoside	C_21_H_22_O_10_	433.1112	−6.5	3
9	12.46	3-Methylellagic acid 8-(2-acetylrhamnoside)	C_23_H_20_O_13_	503.0926	18.9	3
10	13.49	2,3-Di-O-methylellagic acid	C_16_H_10_O_8_	329.0311	2.5	2
11	13.74	6-Methoxytaxifolin	C_16_H_14_O_8_	333.0622	1.8	2
12	16.29	2,3,8-Tri-O-methylellagic acid	C_17_H_12_O_8_	343.0472	3.7	2
13	18.10	Triterpenoid acid, putatively poricoic acid K ^c^	C_30_H_46_O_5_	485.3200	−14.8	3
14	18.74	Acacic acid	C_30_H_48_O_5_	487.3432	0.6	2
15	19.30	Isomer of acacic acid	C_30_H_48_O_5_	487.3438	1.9	4

**^a^** Identification levels according to Schymanski et al. [37]: 1, confirmed with an authentic analytical standard (retention time and MS/MS); 2, probable structure supported by accurate mass (|error| ≤ 5 ppm) and MS/MS product ions; 3, tentative candidate supported by MS/MS product ions but with a precursor mass error above 5 ppm; 4, molecular formula only (MS spectrum, no MS/MS). Errors are relative to the *m*/*z* calculated from the molecular formula. ^b^ Value recorded in the analytical report; incompatible with the assignment by 0.1 Da and most probably a transcription error (461.0707 would correspond to −3.9 ppm). ^c^ The formula is proposed from the accurate mass and MS/MS data but is not supported within the 5 ppm tolerance; the annotation as poricoic acid K is regarded as doubtful (Table S6).

## Data Availability

The original contributions presented in this study are included in the article and its Supplementary Materials. The datasets supporting the conclusions of this article—the archived extraction records (run means and standard deviations of the duplicate extractions), the antioxidant assay readings, the chromatographic raw files and the analytical report, the specimen-level wood-block ratings (Tables S8–S10) and the external laboratory report of the *Daphnia magna bioassays*—will be made available by the corresponding author on reasonable request.

## Supplementary Materials

The following supporting information can be downloaded at: https://www.mdpi.com/article/10.3390/molecules31193503/s1, Table S1: Factors and levels of the 2^3^ factorial design with center point; Table S2: Design matrix in coded and actual units, and crude extract yield on an air-dry bark basis; Table S3: Yield of the fractionated (carbohydrate-depleted) extract after methanol precipitation; Table S4: Antioxidant capacity of the fractionated extracts, expressed as gallic acid equivalents; Table S5: Compounds tentatively identified by GC–MS and their relative abundance; Table S6: Compounds annotated by UHPLC-HR-Q-TOF-MS in negative ionization mode, ordered by retention time, with molecular formula, calculated and observed [M − H]^−^ *m*/*z*, mass error, diagnostic MS/MS product ions and identification level; Table S7: Commercial products used as references and their active ingredients; Table S8: Trial 1—bark extract combined with commercial products; Table S9: Trial 2—natural components applied individually across a concentration range; Table S10: Trial 3—natural components combined with the copper-8-quinolinolate product CP1; Table S11: Test conditions and acceptability criteria; Table S12: 48-h median lethal concentrations (LC_50_) reported by the external laboratory, test items and hazard classification; Table S13: Analysis of the 2^3^ factorial model for the crude extraction yield, tested against the pure-error variance of the duplicate runs; Table S14: Non-parametric analysis of the specimen-level wood-block ratings (Tables S8–S10).

## Abbreviations

The following abbreviations are used in this manuscript:

CCA	Chromated Copper Arsenate
Hk	Hinokitiol
CP1–CP4	Commercial anti-sapstain products 1–4 (CP1, copper-8-quinolinolate, 5.2% *w*/*w*; CP2, chlorothalonil, 72% *w*/*v*; CP3, carbendazim, 50% *w*/*v*; CP4, copper-8-quinolinolate, 5.91% *w*/*w*, positive control of the diffusion assay)
BPR	Biocidal Products Regulation
DPPH	2,2-diphenyl-1-picrylhydrazyl
ABTS	2,2′-azino-bis(3-ethylbenzothiazoline-6-sulfonic acid)
FRAP	Ferric Reducing Antioxidant Power
GAE	Gallic Acid Equivalents
TPTZ	2,4,6-tripyridyl-s-triazine
CLSI	Clinical and Laboratory Standards Institute
ASTM	American Society for Testing and Materials
LC_50_	Median Lethal Concentration
GC-MS	Gas Chromatography–Mass Spectrometry
UHPLC-MS	Ultra-High-Performance Liquid Chromatography–Mass Spectrometry
EBE-S	Selected Eucalyptus Bark Extract (most favourable condition within the tested range)
SD	Standard Deviation
CI	Confidence Interval
ISO	International Organization for Standardization
ANOVA	Analysis of Variance
CytP450	Cytochrome P450 monooxygenases
CBH1	Cellobiose dehydrogenase
BGL1	β-glucosidase
