# Supplementary material for: Eucalyptus globulus Bark Extract and Hinokitiol in Hybrid Anti-Sapstain Formulations with Reduced Chlorothalonil, Carbendazim and Copper-8-quinolinolate Loads
Source: Molecules. 2026 Oct 1;31(19):3503. doi: 10.3390/molecules31193503 (PMC13666255; doi:10.3390/molecules31193503)
Supplement: Supplementary file 1 [file molecules-31-03503-s001.zip › molecules-4554897-supplementary.pdf]

Supplementary Materials

Eucalyptus globulus Bark Extract and Hinokitiol in Hybrid Anti-Sapstain Formulations with Reduced Chlorothalonil, Carbendazim and Copper-8-quinolinolate Loads

Gastón Bravo-Arrepol, Solange Torres, Victor Ferrer, Danilo Escobar-Avello, Moisés Vásquez, Vicente Hernández, Gustavo Cabrera-Barjas, Claudia Pérez and Cecilia Fuentealba

S1. Screening of the extraction conditions

Extraction conditions were screened with a 2^3 full factorial design augmented with a center point (eight factorial runs plus one center run), generated and analyzed in Design-Expert 11. Each run was performed in duplicate on 62.5 g of air-dried bark (moisture content not determined) with 500 mL of solvent in 1 L jacketed reactors; the extract was recovered by vacuum filtration through Whatman No. 1 paper, rotary evaporation at 45 °C and lyophilisation. Yields are expressed relative to the mass of air-dried bark.

Table S1. Factors and levels of the 2^3 factorial design with center point.

Factor	Symbol	Unit	Low (−1)	Center (0)	High (+1)
Extraction temperature	X_1	°C	75	85	95
Ethanol concentration	X_2	% v/v	40	50	60
Extraction time	X_3	h	1	2	3

Table S2. Design matrix in coded and actual units, and crude extract yield on an air-dry bark basis.

Run	X_1	X_2	X_3	T (°C)	Ethanol (% v/v)	t (h)	Yield (%, d.b.)
STD1	−1	−1	−1	75	40	1	5.02 ± 0.57
STD2	−1	+1	−1	75	60	1	5.94 ± 0.29
STD3	−1	−1	+1	75	40	3	5.61 ± 0.04
STD4	−1	+1	+1	75	60	3	4.51 ± 0.08
STD5	0	0	0	85	50	2	5.19 ± 0.34
STD6	+1	+1	−1	95	60	1	5.36 ± 0.07
STD7	+1	−1	−1	95	40	1	4.84 ± 0.09
STD8	+1	+1	+1	95	60	3	4.12 ± 0.47
STD9	+1	−1	+1	95	40	3	4.41 ± 0.40

Values are mean ± standard deviation of duplicate extractions; only these run summaries were archived, and the individual duplicate values used in Table S13 were reconstructed from them (see Table S13). The shaded row is the center point.

Table S3. Yield of the fractionated (carbohydrate-depleted) extract after methanol precipitation.

Run	Crude extract (%, d.b.)	Fractionated extract (%, d.b.)	Recovery (%)
STD1	5.02	3.04	60.6

Run	Crude extract (%, d.b.)	Fractionated extract (%, d.b.)	Recovery (%)
STD2	5.94	4.23	71.2
STD3	5.61	3.45	61.5
STD4	4.51	3.53	78.3
STD5	5.19	3.76	72.4
STD6	5.36	3.59	67.0
STD7	4.84	3.48	71.9
STD8	4.12	3.56	86.4
STD9	4.41	3.14	71.2

Recovery is calculated as fractionated yield divided by crude yield. Residual carbohydrates remained in every fractionated extract (Table 1 of the main text).

Table S4. Antioxidant capacity of the fractionated extracts, expressed as gallic acid equivalents.

Run	DPPH (mg GAE g^{-1})	ABTS (mg GAE g^{-1})	FRAP (mg GAE g^{-1})
STD1	77.32	80.7	125.4
STD2	72.72	88.7	126.2
STD3	65.38	88.6	122.9
STD4	78.04	93.5	134.3
STD5	75.68	91.7	131.4
STD6	85.52	103.0	141.1
STD7	81.96	88.9	130.4
STD8	88.23	106.8	138.0
STD9	82.45	90.7	129.5

Values are the means of the two independent extractions of each run (each extraction measured in technical triplicate; 96-well microplate format, 25 µL of extract at 1 mg mL^{-1} plus 250, 225 and 250 µL of DPPH, ABTS•+ and FRAP reagent, respectively; gallic acid calibration 1–40, 1–20 and 1–200 mg L^{-1}); differences among treatments were not statistically significant in any of the three assays (one-way ANOVA and Tukey's HSD on the extraction means, n = 2 per treatment, $p > 0.05$). Whether the fractionation step concentrated the bioactive compounds cannot be established from these data, because a paired crude-versus-fractionated comparison of the same extraction was not performed. The shaded row is the center point.

S2. Chemical characterization of the extract

Table S5. Compounds tentatively identified by GC–MS and their relative abundance.

Peak	RT (min)	Compound	Relative abundance (%)
1	5.61	1,3-Di-tert-butylbenzene	3.63
2	8.32	Aromadendrene	2.50
3	9.21	2,5-Di-tert-butylphenol	0.77
4	9.67	Mellein	1.65

Peak	RT (min)	Compound	Relative abundance (%)
5	9.87	Globulol	1.23
6	10.18	*γ*-Gurjunene	5.58
7	10.53	3,4,5-Trimethoxyphenol	0.59
8	10.91	*α*-Eudesmol	0.58
9	15.45	Methyl oleate	1.13
10	26.85	*β*-Sitosterol	37.29
11	27.34	Amyrin	20.78
		**Total identified**	75.73

Non-derivatised extracts; Agilent 7890A/5975C, splitless injection at 250 °C, HP-5MS column, helium at 1 mL min^{-1}, oven 60–300 °C at 10 °C min^{-1}, electron ionisation at 70 eV, m/z 50–550. Identification was based solely on comparison of the mass spectra with the NIST 05 library (match > 90%) and published spectra; no authentic standards were analysed and retention indices were not calculated, so all assignments, including β-sitosterol and amyrin, are tentative. 1,3-Di-tert-butylbenzene and 2,5-di-tert-butylphenol are regarded as probable process contaminants. Peak numbers correspond to the annotated chromatogram. Abundances are percentages of total ion current.

Table S6. Compounds annotated by UHPLC-HR-Q-TOF-MS in negative ionization mode, ordered by retention time, with molecular formula, calculated and observed $[M-H]^-$ m/z, mass error, diagnostic MS/MS product ions and identification level.

No.	RT (min)	Putative compound	Formula	Calc. m/z	Obs. m/z	Error (ppm)	MS/MS product ions (m/z)	Level
1	1.70	Quinic acid	$C_7H_{12}O_6$	191.0561	191.0557	−2.2	155.0, 127.0, 111.0	1
2	3.56	1-Galloyl-β-D-glucose	$C_{13}H_{16}O_{10}$	331.0671	331.0687	4.9	271.0, 211.0, 169.0, 151.0, 125.0	2
3	7.32	Ellagic acid glucoside	$C_{20}H_{16}O_{13}$	463.0518	463.0521	0.6	343.0, 315.0, 299.9	2
4	8.53	Trimethoxyphenyl-galloyl-glucopyranoside	$C_{22}H_{26}O_{13}$	497.1301	497.1267	−6.8	313.0, 211.0, 169.0, 153.0, 125.0	3
5	8.70	Erythroxyloside B	$C_{21}H_{30}O_{13}$	489.1614	489.1580	−6.9	387.1, 345.1, 243.0, 183.0, 153.0, 125.0	3
6	9.36	Isoquercitrin	$C_{21}H_{20}O_{12}$	463.0882	463.0848	−7.3	419.1, 359.1, 300.0, 271.0, 255.0, 243.0	1
7	10.50	Kaempferol 7-O-glucuronide	$C_{21}H_{18}O_{12}$	461.0725	461.1707 [a]	—	315.0, 299.9, 270.9	3
8	11.20	Naringenin 7-O-glucoside	$C_{21}H_{22}O_{10}$	433.1140	433.1112	−6.5	286.0, 269.0, 178.9, 152.0, 139.0, 124.0	3
9	12.46	3-Methylellagic acid 8-(2-acetylrhamnoside)	$C_{23}H_{20}O_{13}$	503.0831	503.0926	18.9	443.0, 315.0, 297.9, 269.9, 241.9	3
10	13.49	2,3-Di-O-methylellagic acid	$C_{16}H_{10}O_8$	329.0303	329.0311	2.5	314.0, 270.9, 242.9, 214.9, 187.0, 159.0	2
11	13.74	6-Methoxytaxifolin	$C_{16}H_{14}O_8$	333.0616	333.0622	1.8	318.0, 303.0, 287.9, 259.9, 232.0, 188.0	2
12	16.29	2,3,8-Tri-O-methylellagic acid	$C_{17}H_{12}O_8$	343.0459	343.0472	3.7	328.0, 297.9, 285.0, 269.9, 241.9, 213.9	2
13	18.10	Triterpenoid acid, putatively poricoic acid K [b]	$C_{30}H_{46}O_5$	485.3272	485.3200	−14.8	333.9, 279.0, 160.9	3
14	18.74	Acacic acid	$C_{30}H_{48}O_5$	487.3429	487.3432	0.6	474.3, 473.3, 439.3	2

No.	RT (min)	Putative compound	Formula	Calc. m/z	Obs. m/z	Error (ppm)	MS/MS product ions (m/z)	Level
15	19.30	Isomer of acacic acid	$C_{30}H_{48}O_5$	487.3429	487.3438	1.9	— (MS only)	4

Calculated m/z values are derived from the molecular formula of the deprotonated ion; observed m/z values and product ions are those recorded in the analytical report (UHPLC-HR-Q-TOF-MS, Bruker Impact II; MetaboScape 3.0, SmartFormula3D 5 ppm, MetFrag/PubChem). Identification levels according to Schymanski et al. (Environ. Sci. Technol. 2014, 48, 2097–2098, doi:10.1021/es5002105; ref. [36] of the main text): 1, structure confirmed with an authentic analytical standard (retention time and MS/MS; Merck Millipore); 2, probable structure supported by accurate mass (|error| ≤ 5 ppm) and MS/MS product ions; 3, tentative candidate supported by MS/MS product ions but with a precursor mass error above 5 ppm; 4, molecular formula only (MS spectrum, no MS/MS). Level-3 annotations are not used to support any mechanistic statement. [a] The observed value recorded in the report is incompatible with the assignment by 0.1 Da and is most probably a transcription error (461.0707 would correspond to −3.9 ppm); the annotation is retained as tentative on the basis of its product ions (kaempferol aglycone, m/z 315/300/271). [b] The molecular formula given in the analytical report ($C_{31}H_{48}O_7$) is incompatible with the observed m/z; the formula shown is derived from the accurate mass, and the annotation as poricoic acid K, a triterpenoid acid reported mainly from Wolfiporia cocos, is regarded as doubtful. Formosinoside, listed in an earlier version of this table, is not present in the analytical report and has been removed.

S3. Wood-block trials (ASTM D4445-10)

Pinus radiata D. Don sapwood specimens (7 × 20 × 70 mm, thickness × width × length; moisture content above 40%), cut from knot-free, stain-free sapwood battens of a single tree and of the same growth-ring region and conditioned as prescribed by ASTM D4445-10, were dipped for 10 s in each treatment solution (lyophilized fractionated extract at the stated % m/v in ethanol–water, 20:80 v/v; hinokitiol (% m/v); commercial products at the stated % v/v as supplied), aired for 24 h under sterile conditions, inoculated with a mixed spore suspension of *Trichoderma* sp., *Ophiostoma* sp. and *Diplodia* seriata (strains TRCCP_01, OPCCP_01 and DSCCP_01, respectively; Centro de Biotecnología, Universidad de Concepción; isolated from P. radiata wood or wood products), and incubated at 25 °C and 70–80% relative humidity in darkness for four weeks. Each treatment was applied to ten replicate specimens. The total fungal growth of the mixed inoculum on each specimen was rated visually once on a 0–5 scale of surface coverage (0, no detectable growth; 1, ≤ 20% of the surface; 2, 20–40%; 3, 40–60%; 4, 60–80%; 5, uniform growth over the whole surface; 0.5-unit steps for intermediate cases); because the three fungi were co-inoculated, no fungus-specific ratings exist. The scale is ordinal, so the inhibition percentages derived from it are descriptive indices; inference is based on the ratings themselves (Table S14). The Inhibition (%) column is calculated as (1 − mean treatment rating / mean untreated-control rating) × 100; the Mean ± SD (%) column reports the mean and standard deviation of the per-specimen inhibition (n = 10), which are the values used for the non-parametric analysis described in Section 2.9 of the main text (Table S14). The three trials were independent, with freshly prepared inocula and treatment solutions; trials 1 and 2 were carried out approximately two months apart.

Table S7. Commercial products used as references and their active ingredients.

Product	Active ingredient	Active content	Share in mill mixture
CP1 (commercial product 1)	Copper-8-quinolinolate	5.2% w/w	4%
CP2 (commercial product 2)	Chlorothalonil	72% w/v	0.5%
CP3 (commercial product 3)	Carbendazim	50% w/v	0.5%

CP1–CP3 are the commercial anti-sapstain products used by the collaborating sawmill, identified by their declared active ingredients (trade names are not given; Section 2.1 of the main text). Percentages in Tables S8–S10 refer to the commercial product

as supplied (% v/v), not to the active ingredient. The mixture used by the sawmill is 4% CP1, 0.5% CP2 and 0.5% CP3 (v/v). Densities at 20 °C: CP1 1.05 g cm^{-3}, CP2 1.08–1.12 g cm^{-3}, CP3 1.162 g cm^{-3}. Active-ingredient loads per 100 mL of treatment solution, calculated from the v/v product doses and the declared contents: industrial reference (4/0.5/0.5% v/v) — copper-8-quinolinolate 4 × 1.05 × 0.052 = 0.218 g, chlorothalonil 0.5 × 0.72 = 0.360 g, carbendazim 0.5 × 0.50 = 0.250 g, total 0.828 g; hybrid formulations with 2% and 1% CP1 — 0.109 and 0.055 g of copper-8-quinolinolate and no chlorothalonil or carbendazim, i.e., reductions of 86.8% and 93.4% in synthetic active ingredients.

Table S8. Trial 1 — bark extract combined with commercial products.

Treatment	Bark extract	CP1	CP2	CP3	Inhibition (%)	Mean ± SD (%)
1	—	4%	0.50%	0.50%	100	100 ± 0.0
2	10%	—	—	—	33	33 ± 10.6
3a	5%	2%	0.25%	0.25%	100	100 ± 0.0
3b	5%	—	0.25%	—	47	47 ± 6.7
4	5%	—	—	0.25%	87	87 ± 8.2
5	5%	—	0.25%	0.25%	93	93 ± 4.8
6	5%	2%	0.25%	—	100	100 ± 0.0
7	—	2%	—	0.25%	100	100 ± 0.0
8	—	2%	0.25%	0.25%	93	93 ± 4.8
Control	—	—	—	—	0	0 ± 0.0

Bark extract alone at 10% gave 33% inhibition in this trial (40% in trial 2, Table S9; the difference is attributed to between-trial variability). Every mixture containing at least 2% CP1 reached 100%.

Table S9. Trial 2 — natural components applied individually across a concentration range.

Component	Concentration (%)	Inhibition (%)	Mean ± SD (%)
Hinokitiol	0.025	64	64 ± 5.2
	0.05	73	73 ± 6.7
	0.1	82	82 ± 4.2
	0.2	91	91 ± 8.8
	0.4	100	100 ± 0.0
Bark extract	0.625	9	9 ± 3.2
	1.25	9	9 ± 5.7
	2.5	27	27 ± 4.8
	5	39	39 ± 5.7
	10	40	40 ± 4.7
Hinokitiol + Bark extract	0.4 / 5	100	100 ± 0.0
Untreated control	—	0	0 ± 0.0

Hinokitiol was dose-dependent and reached complete inhibition at 0.4%. The bark extract did not exceed 40% inhibition even at the highest concentration tested.

Table S10. Trial 3 — natural components combined with the copper-8-quinolinolate product CP1.

**Formulation**	**Inhibition (%)**	**Mean ± SD (%)**
Hinokitiol 0.1% / CP1 1%	93	93 ± 11.6
Hinokitiol 0.1% / CP1 2%	100	100 ± 0.0
Hinokitiol 0.2% / CP1 1%	90	90 ± 9.4
Hinokitiol 0.2% / CP1 2%	97	97 ± 9.5
Bark extract 5% / CP1 1%	93	93 ± 10.6
Bark extract 5% / CP1 2%	97	97 ± 6.7
Hinokitiol 0.4% / Bark extract 5%	95	95 ± 8.5
Hinokitiol 0.4% (control)	100	100 ± 0.0
CP1 2% (control)	100	100 ± 0.0
CP1 4% / CP2 0.5% / CP3 0.5% (synthetic reference; industrial mixture)	100	100 ± 0.0
Untreated control	0	0 ± 0.0

In this laboratory trial, formulations in which CP2 and CP3 were omitted and replaced by bark extract at 5% or hinokitiol at 0.1–0.2% maintained inhibition at or above 90% with CP1 at 1–2%, i.e., at half the CP1 dose used industrially (4%). Because CP1 at 2% alone also gave 100% inhibition and no CP1 1%-only control was included, the incremental contribution of the natural components cannot be isolated from these data. The rating of one specimen of the hinokitiol 0.2% / CP1 1% treatment was recorded as 0.1 in the raw file and has been corrected to 0 (the scale runs in 0.5-unit steps); the SD reported for that treatment (9.4) reflects the corrected value. The non-parametric analysis of these ratings is given in Table S14.

S4. Acute toxicity to Daphnia magna

The bioassays were commissioned to an external environmental-testing laboratory (ECOMBIO Ltda., Concepción, Chile; report no. 03082022_UDT04, 3 August 2022) and performed according to the Chilean standards NCh 2083 Of.1999 and NCh 2706 Of.2002, which are harmonised with ISO 6341. For each test item a range-finding test was followed by a definitive test with five concentrations in geometric progression from the undiluted test item (100%) plus a dilution-water control (0%). The test items were the selected fractionated bark extract (EBE-S), hinokitiol and the copper-8-quinolinolate product CP1 tested alone as the synthetic reference; chlorothalonil and carbendazim products were not tested. Mortality was recorded after 24 and 48 h and the 48-h median lethal concentration (LC50) was estimated by the laboratory; chamber-level counts, the fitting model and confidence intervals were not part of the laboratory report and are therefore not available (Table S12).

Table S11. Test conditions and acceptability criteria.

**Parameter**	**Condition**
Test type	Static, without renewal
Test duration	24 and 48 h
Temperature	20 ± 0.2 °C
Photoperiod	16 h light / 8 h dark
Test chamber volume	25 mL
Test solution volume	20 mL

Parameter	Condition
Age of organisms	Neonates < 24 h
Organisms per chamber	5
Replicates per concentration	4
Organisms per concentration	20
Dilution water	Reconstituted water, hardness 250 mg L^{-1} $CaCO_3$, pH 7.8
Endpoint	Mortality (48-h LC_{50})
Acceptability criterion	Control survival > 90%; dissolved oxygen > 80% saturation

Table S12. 48-h median lethal concentrations (LC50) reported by the external laboratory, test items and hazard classification.

Test item	Description	48-h LC50 (ppm, nominal)	Hazard class [a]	Remarks
EBE-S	Lyophilized fractionated bark extract (STD2 conditions)	27 500	Low toxicity	Five geometric dilutions; 4 chambers × 5 neonates per concentration
CP1 (commercial product 1)	Copper-8-quinolinolate product (5.2% w/w), tested alone as synthetic reference	38.71	Medium toxicity	≈ 2.0 ppm copper-8-quinolinolate on an active-ingredient basis
Hinokitiol	β-Thujaplicin, > 98%	Not reported	—	The concentration range required to reach 50% mortality exceeded the aqueous solubility of hinokitiol (≈ 1.2 g L^{-1}); the nominal result was outside the workable scale of the assay (main text, Section 3.5)

Values are the point estimates reported by ECOMBIO Ltda. (report 03082022_UDT04) under NCh 2083 Of.1999 and NCh 2706 Of.2002; endpoint, mortality after 48 h of static exposure. Chamber-level counts, the concentration–response model and 95% confidence intervals were not provided in the laboratory report and are reported here as a limitation. [a] *Classification according to Circular D.G.T.M. y M.M. Ordinario N° A-52/008 (Chilean maritime authority), as applied by the laboratory. On a product-mass basis the extract was approximately 710-fold less acutely toxic than CP1; this ratio refers to the copper product alone and not to the complete industrial mixture.*

Table S13. Analysis of the 2^3 factorial model for the crude extraction yield, tested against the pure-error variance of the duplicate runs.

Source / term	Coefficient (coded)	Sum of squares	df	Mean square	F	p
Model (factorial terms + curvature)	—	5.6200	8	0.7025	6.82	0.0047
Intercept (factorial mean)	4.9763	—	—	—	—	—
X_1 (temperature)	−0.2938	1.3806	1	1.3806	13.41	0.0052
X_2 (ethanol)	+0.0063	0.0006	1	0.0006	0.01	0.9396
X_3 (time)	−0.3138	1.5750	1	1.5750	15.30	0.0036
X_1X_2	+0.0513	0.0420	1	0.0420	0.41	0.5388
X_1X_3	−0.1037	0.1722	1	0.1722	1.67	0.2281
X_2X_3	−0.3538	2.0022	1	2.0022	19.45	0.0017
$X_1X_2X_3$	+0.1512	0.3660	1	0.3660	3.56	0.0920

Source / term	Coefficient (coded)	Sum of squares	df	Mean square	F	p
Curvature (centre vs. factorial mean)	+0.2138	0.0812	1	0.0812	0.79	0.3975
Pure error (residual)	—	0.9265	9	0.1029	—	—
Total (corrected)	—	6.5465	17	—	—	—

Analysis of the 18 observations (nine runs × two extractions). Coefficients are in coded units ($X_1 = (T - 85)/10$; X_2 = (ethanol concentration − 50)/10; $X_3 = (t - 2)/1$); the effect of each factor equals twice its coefficient. All model terms are orthogonal, so the sum of squares of each factorial term equals 16 × coefficient², and every coefficient has the same standard error, SE = √(MS pure error/16) = 0.0802. Pure-error mean square = 0.1029 (9 df, one per duplicated run); $R^2 = 0.858$ and adjusted $R^2 = 0.733$ for the model including the curvature term ($R^2 = 0.872$ for the seven factorial terms fitted to the 16 factorial observations); root mean square error = 0.321% yield. Temperature (X_1), time (X_3) and the ethanol × time interaction (X_2X_3) were significant at $p < 0.05$, whereas the ethanol main effect was not. The centre point gave no evidence of curvature within the tested ranges ($F(1,9) = 0.79$, $p = 0.40$), supporting a first-order model with interaction terms. Residual diagnostics: the residuals were symmetrically distributed with no trend against fitted values or run order, and the Shapiro–Wilk test did not reject normality ($W = 0.969$, $p = 0.78$). Limitation: only the mean ± SD of the duplicate extractions of each run was archived, so the two replicate values per run were reconstructed as mean ± SD/√2 before analysis. For n = 2 this reproduces the run means and the pure-error sum of squares exactly, but it relies on rounded summary values, which may slightly alter the exact p values, and it fixes the within-run spread by construction, so a formal homogeneity-of-variance test on the residuals is not informative; the run standard deviations ranged from 0.04% (STD3) to 0.57% (STD1).

Table S14. Non-parametric analysis of the specimen-level wood-block ratings (Tables S8–S10).

Test / contrast	Data	Statistic	p (raw)	p (Holm)	Hodges–Lehmann difference in inhibition, points (95% CI)
Kruskal–Wallis, trial 1 (10 treatments)	Table S8, n = 10 per treatment	H = 89.0, df = 9	< 0.001	—	—
Kruskal–Wallis, trial 2 (12 treatments)	Table S9	H = 116.3, df = 11	< 0.001	—	—
Kruskal–Wallis, hinokitiol dose series (5 levels)	Table S9	H = 41.8, df = 4	< 0.001	—	—
Kruskal–Wallis, bark-extract dose series (5 levels)	Table S9	H = 43.7, df = 4	< 0.001	—	—
Jonckheere–Terpstra trend, hinokitiol 0.025–0.4% (n = 50)	Table S9	JT = 920, z = 7.41	< 0.001	—	—
Jonckheere–Terpstra trend, bark extract 0.625–10% (n = 50)	Table S9	JT = 892, z = 6.96	< 0.001	—	—
Kruskal–Wallis, trial 3 (11 treatments)	Table S10	H = 60.5, df = 10	< 0.001	—	—
C1: Hk 0.4% + EBE 5% vs Hk 0.4% alone	Trial 3; 95 vs 100	U = 35	0.078	0.39	0 (−10 to 0)
C2: EBE 5% + CP1 2% vs CP1 2% alone	Trial 3; 97 vs 100	U = 40	0.168	0.50	0 (0 to 0)
C3: Hk 0.2% + CP1 2% vs CP1 2% alone	Trial 3; 97 vs 100	U = 45	0.368	0.74	0 (0 to 0)
C4: Hk 0.1% + CP1 2% vs CP1 2% alone	Trial 3; 100 vs 100	U = 50	1.000	1.00	0 (0 to 0)
C5: EBE 5% + CP1 1% vs synthetic reference	Trial 3; 93 vs 100	U = 30	0.035	0.21	0 (−10 to 0)
C6: Hk 0.2% + CP1 1% vs synthetic reference	Trial 3; 90 vs 100	U = 20	0.006	0.040	−10 (−20 to 0)
C7: Hk 0.1% + CP1 1% vs synthetic reference	Trial 3; 93 vs 100	U = 35	0.078	0.39	0 (−20 to 0)
E1 (exploratory, between trials): EBE 5% + CP1 1% (trial 3) vs EBE 5% alone (trial 2)	93 vs 39	U = 100	< 0.001	< 0.001	+60 (50 to 60)

Test / contrast	Data	Statistic	p (raw)	p (Holm)	Hodges–Lehmann difference in inhibition, points (95% CI)
E2 (exploratory, between trials): Hk 0.2% + CP1 1% (trial 3) vs Hk 0.2% alone (trial 2)	90 vs 91	U = 47	0.840	0.84	0 (−10 to 10)
T1: EBE 5% + CP2 0.25% + CP3 0.25% vs EBE 5% + CP2 0.25%	Trial 1; 93 vs 47	U = 100	< 0.001	< 0.001	+50 (40 to 50)
T2: EBE 5% + CP3 0.25% vs EBE 5% + CP2 0.25%	Trial 1; 87 vs 47	U = 100	< 0.001	< 0.001	+40 (30 to 50)

The experimental unit is the individual specimen (n = 10 per treatment; one 0–5 rating of total fungal growth per specimen). Kruskal–Wallis tests were run within each trial on the ratings; Jonckheere–Terpstra tests (normal approximation with tie correction) tested the monotonic increase of inhibition with concentration across the five levels of each dose series (5 × 10 = 50 specimens). Pairwise contrasts C1–C7 were pre-specified within trial 3 and use the two-sided asymptotic Mann–Whitney U test (n = 10 + 10, ties corrected) with Holm adjustment within that family; T1–T2 form a second family within trial 1. E1 and E2 compare a natural component applied alone in trial 2 with its combination with 1% CP1 in trial 3 and are reported as exploratory because the observations are not protected by a common trial. Hodges–Lehmann estimates are the median of the pairwise differences in per-specimen inhibition (first-named minus second-named treatment) with a 95% confidence interval from the Mann–Whitney distribution; because ratings are discrete (0.5-unit steps) and many specimens scored 0, intervals are conservative and several collapse to zero width. The absence of a detectable difference is not interpreted as equivalence. Analyses were performed in Python 3.11 with SciPy.
